# Mapping Atmospheric Moisture Climatologies across the Conterminous United States

**DOI:** 10.1371/journal.pone.0141140

**Published:** 2015-10-20

**Authors:** Christopher Daly, Joseph I. Smith, Keith V. Olson

**Affiliations:** PRISM Climate Group, Northwest Alliance for Computational Science and Engineering, Oregon State University, Corvallis, Oregon, United States of America; University of Colorado, UNITED STATES

## Abstract

Spatial climate datasets of 1981–2010 long-term mean monthly average dew point and minimum and maximum vapor pressure deficit were developed for the conterminous United States at 30-arcsec (~800m) resolution. Interpolation of long-term averages (twelve monthly values per variable) was performed using PRISM (Parameter-elevation Relationships on Independent Slopes Model). Surface stations available for analysis numbered only 4,000 for dew point and 3,500 for vapor pressure deficit, compared to 16,000 for previously-developed grids of 1981–2010 long-term mean monthly minimum and maximum temperature. Therefore, a form of Climatologically-Aided Interpolation (CAI) was used, in which the 1981–2010 temperature grids were used as predictor grids. For each grid cell, PRISM calculated a local regression function between the interpolated climate variable and the predictor grid. Nearby stations entering the regression were assigned weights based on the physiographic similarity of the station to the grid cell that included the effects of distance, elevation, coastal proximity, vertical atmospheric layer, and topographic position. Interpolation uncertainties were estimated using cross-validation exercises. Given that CAI interpolation was used, a new method was developed to allow uncertainties in predictor grids to be accounted for in estimating the total interpolation error. Local land use/land cover properties had noticeable effects on the spatial patterns of atmospheric moisture content and deficit. An example of this was relatively high dew points and low vapor pressure deficits at stations located in or near irrigated fields. The new grids, in combination with existing temperature grids, enable the user to derive a full suite of atmospheric moisture variables, such as minimum and maximum relative humidity, vapor pressure, and dew point depression, with accompanying assumptions. All of these grids are available online at http://prism.oregonstate.edu, and include 800-m and 4-km resolution data, images, metadata, pedigree information, and station inventory files.

## Introduction

The demand for spatial climate data sets in digital form continues to increase, as more and more climate-driven modeling and analysis activities are performed within spatially-explicit computing environments. Key inputs to these analyses are grids of thirty-year decadal climate averages (e.g., 1971–2000, 1981–2010, etc.), termed “normals,” that describe the values and spatial patterns that can be expected in an average year or month. Through various forms of a technique called Climatologically-Aided Interpolation (CAI; see Mapping Methods section), climate normal grids also provide the foundation for a number of spatially distributed climate products that cover individual years, months and days [1–4]. Normals produced by the PRISM (Parameter-elevation Relationships on Independent Slopes Model) Climate Group at Oregon State University are often used for this purpose [5–9].

Most spatial climate normals are limited to temperature and precipitation, but measures of atmospheric moisture content and deficit are also important in a variety of processes, including plant function [10–14], drought severity and wildfire behavior [15], [16], and climate change impacts [17], [18]. Relatively little work has been to done to produce long-term normal grids of such variables, partly due to a lack of observations. Empirical relationships have been developed to derive humidity measures from available temperature and precipitation data (e.g., [19–21]), but such methods incorporate assumptions that are violated in certain regions (e.g., [22]). Actual observations of mean dew point (*T*_*dmean*_) have been included in monthly time series datasets ([9]), which in turn have been incorporated into other datasets (e.g., [23]), but there has been little focus on long-term normals of *T*_*dmean*_. Further, long-term normals of atmospheric moisture deficit have received even less attention. A useful variable in this regard is vapor pressure deficit (*VPD*), which is the difference between the saturation vapor pressure (dictated by temperature alone) and the actual vapor pressure. *VPD* is a direct measure of the deficit, unlike relative humidity (*RH)*, for which one must know the temperature at the time of measurement [24]. Ideally, one would want to know the average minimum *VPD* (*VPD*_*min*_) and maximum *VPD* (*VPD*_*max*_), which bracket the range of moisture deficit conditions throughout an average day. This is similar to the advantage of knowing the average maximum and minimum temperature as opposed to mean temperature only. An added advantage is that *T*_*dmean*_, *VPD*_*min*_ and *VPD*_*max*_, in combination with existing grids of minimum and maximum temperature (*T*_*min*_ and *T*_*max*_), allow many other atmospheric moisture variables to be derived, such as minimum and maximum *RH*, vapor pressure, and dew point depression, with accompanying assumptions (see Relative Humidity Derivation section, for example).

This paper describes the development of spatial climate normals of 1981–2010 mean monthly *T*_*dmean*_, *VPD*_*min*_ and *VPD*_*max*_ across the conterminous United States, using methods that strive to account for the major physiographic factors influencing climate patterns. The *T*_*dmean*_ normals are updates of datasets used in the development of a century-long monthly time series [9]. The *VPD*_*min*_ and *VPD*_*max*_ datasets are, to our knowledge, the first of their kind, although datasets of mean *RH* have been developed [25]. Section 2 of this paper describes the study area, preparation of station data, and mapping methods. Section 3 presents the resulting gridded data sets, and discusses model performance. Concluding remarks are given in Section 4.

## Study Area and Work Flow

**Fig 1:**
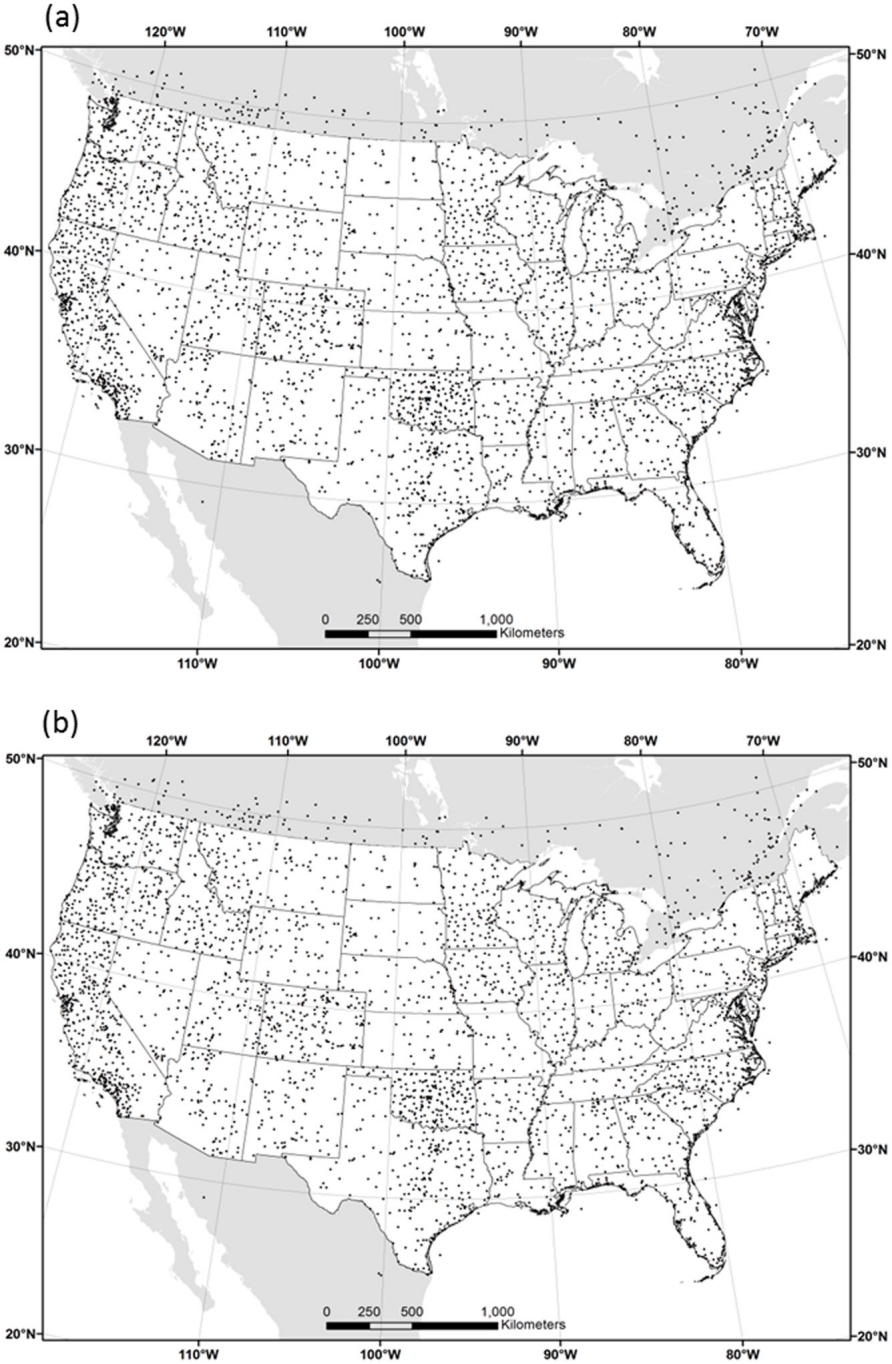
Map of study area and station locations. Conterminous US and border area monthly average (a) mean dew point (*T*_*dmean*_) stations and (b) minimum and maximum vapor pressure deficit (*VPD*_*min*_ and *VPD*_*max*_) stations.

Climate data sets were developed for the conterminous US at 30 arc-second resolution in geographic (latitude/longitude) coordinates (Fig 1). A 30-arc-second grid cell is approximately 900 x 700 m at 40N latitude, and is referred to here as “800 m.” The boundaries of the grid are 22°N and 50°N and 65°W and 125°W, which are exactly coincident with other 800-m grids from the PRISM Climate Group [8]. The interpolation was performed separately in three overlapping regions: western, central and eastern US, and the resulting grids merged to form a complete conterminous US grid. The western region extends from the Pacific Coast to eastern Colorado, central from central Colorado to Lake Michigan, and eastern from eastern Minnesota to the eastern seaboard.

Care was taken to include as many islands offshore the US mainland as possible, but undoubtedly some very small islands were missed. To accommodate GIS shoreline data sets of varying quality and resolution, the modeling region was extended offshore several km and generalized to include bays and inlets. However, the gridded climate estimates are valid over land areas only.

**Fig 2:**
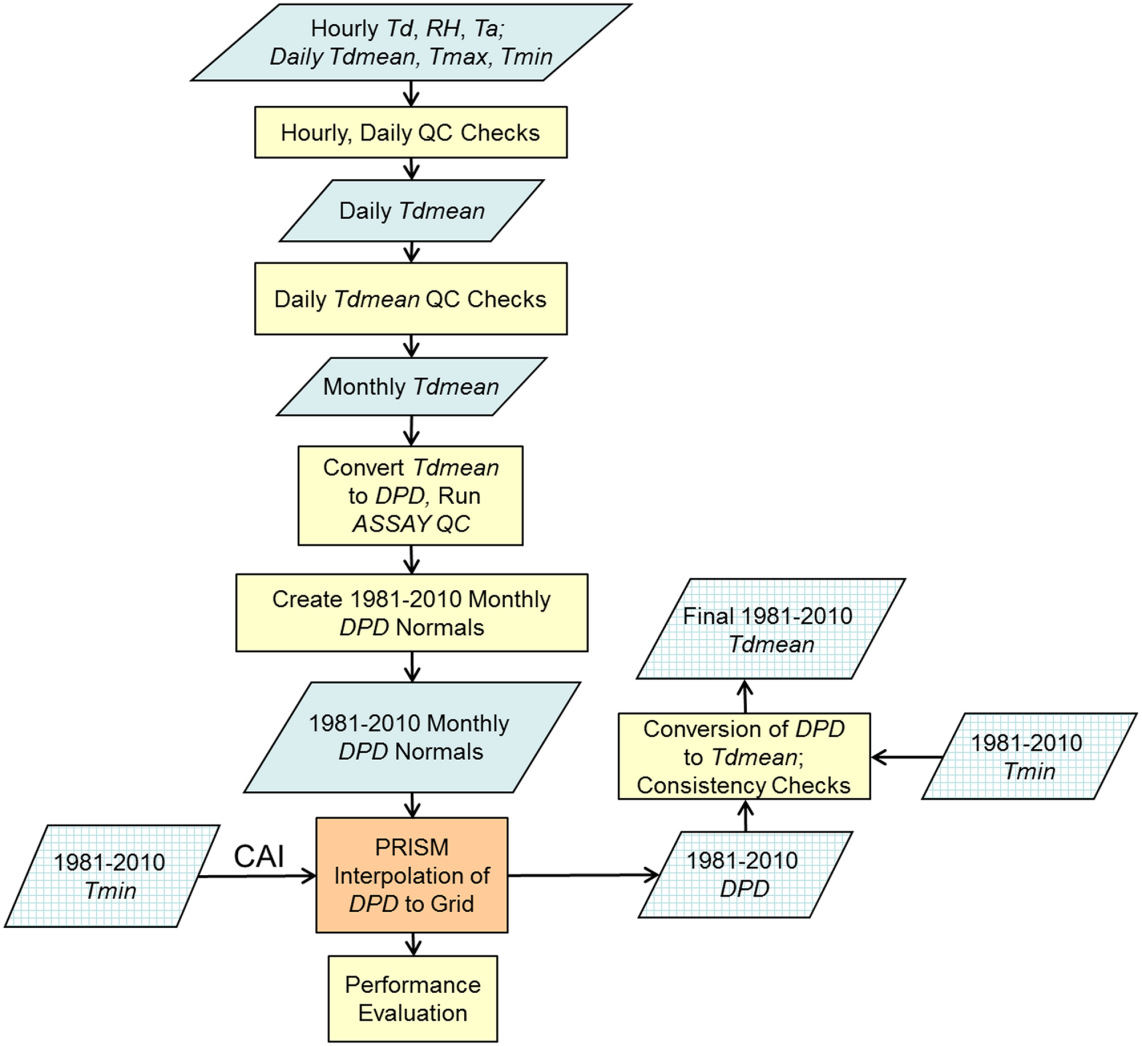
Work flow diagram for data processing and mapping of *T*_*dmean*_. Cross-hatched boxes represent gridded data. See text for abbreviation definitions.

**Fig 3:**
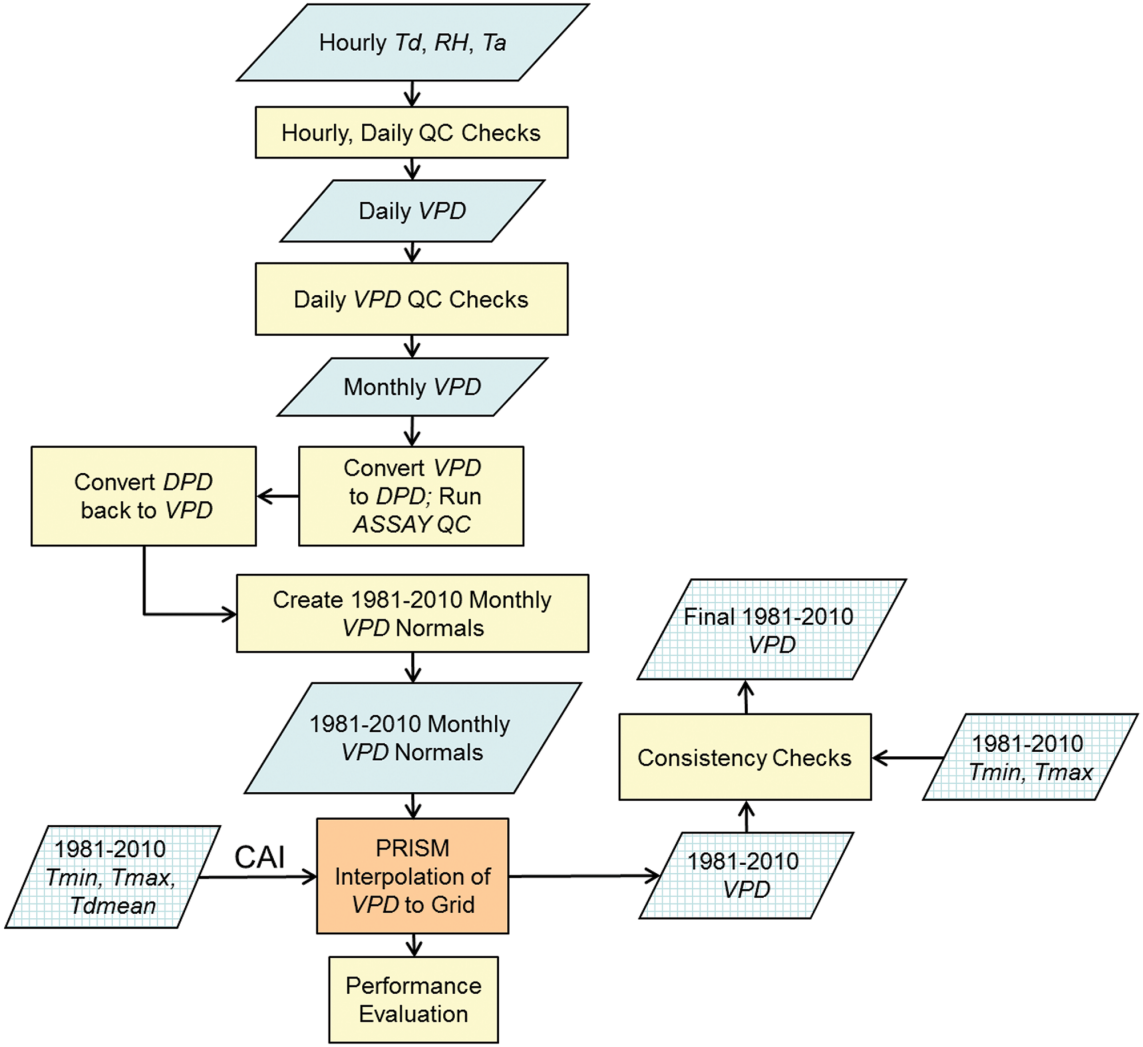
Work flow diagram for data processing and mapping of *VPD*_*min*_ and *VPD*_*max*_. *VPD* refers to either *VPD*_*min*_ or *VPD*_*max*_. Cross-hatched boxes represent gridded data. See text for abbreviation definitions.

Overviews of the data processing and mapping work flows for *T*_*dmean*_ and for *VPD*_*min*_ and *VPD*_*max*_ are diagrammed in Figs 2 and 3, respectively. The process began with hourly and daily data for *T*_*dmean*_, and hourly data for *VPD*_*min*_ and *VPD*_*max*_, which were averaged to daily, and then monthly, time steps, with quality screening done at each stage (see Station Data and Processing section). At the monthly time step, all three variables were expressed as dew point depression (*DPD*) to take advantage of a spatial consistency quality control (QC) method using a version of PRISM called ASSAY (see Quality Control and Calculation of Monthly Values section). *T*_*dmean*_ was kept in the form of *DPD* for interpolation by PRISM, and converted back to *T*_*dmean*_ as a grid post-processing step. *VPD*_*min*_ and *VPD*_*max*_ were converted from *DPD* back to their original forms before interpolation by PRISM (see Mapping Methods section). A performance evaluation was conducted on the PRISM interpolation process (see Uncertainty Analysis section). As a final step, output grids from PRISM were checked for consistency with previously-created grids of 1981–2010 *T*_*max*_ and *T*_*min*_.

## Station Data and Processing

### Data Sources

Data from surface stations, numbering about 4,000 for *T*_*dmean*_ (44 km average station spacing) and 3,500 for *VPD*_*max*_ and *VPD*_*min*_ (47 km average station spacing) were obtained from a variety of sources (Table 1; Fig 1). Data from the National Weather Service (NWS) Automated Surface Observing System (ASOS) came via Unidata's Internet Data Distribution system and the National Climatic Data Center (NCDC) Integrated Surface Hourly/Integrated Surface Data archives (ftp://ftp.ncdc.noaa.gov/pub/data/noaa/), supplemented by the Solar And Meteorological Observation Network (SAMSON) and Integrated Surface Weather Observations CD-ROMs. NWS Cooperative Observer Program (COOP) and Weather Bureau Army Navy (WBAN) data were obtained from (http://www.ncdc.noaa.gov/cdo-web/). USDA Forest Service and Bureau of Land Management Remote Automatic Weather Station (RAWS) data came from archives at the Western Regional Climate Center (http://www.raws.dri.edu), the Real-time Observation Monitor and Analysis Network http://raws.wrh.noaa.gov), and from the MesoWest Local Data Manager (LDM) feed and website (http://mesowest.utah.edu/data). USDA Natural Resources Conservation Service (NRCS) Soil Climate Analysis Network (SCAN) observations were provided through the National Water and Climate Center web service (http://www.wcc.nrcs.usda.gov). Bureau of Reclamation AgriMet (AGRIMET) data were obtained from the Pacific Northwest Region (http://www.usbr.gov/pn/agrimet/), Great Plains Region (http://www.usbr.gov/gp/agrimet/), Upper Colorado Region in cooperation with the Utah Climate Center (https://climate.usurf.usu.edu/agweather.php), and Mid Pacific Region in cooperation with the Desert Research Institute (http://nicenet.dri.edu/) and the National Oceanic and Atmospheric Administration NOAA Idaho National Laboratory Weather Center (http://niwc.noaa.inel.gov/). Data were also collected from the Oklahoma Climatological Survey Mesonet (OKMESONET; http://www.mesonet.org/data/public/mesonet/mts), Colorado State University’s Colorado Agricultural Meteorological Network (COAGMET; http://ccc.atmos.colostate.edu/~coagmet), and a small number of miscellaneous stations from state and local networks.

**Table 1:** Source, averaging interval, and number of stations used in the mapping process.

Source	Averaging Interval	Stations Contributing ≥ 1 Month to Grids		Stations Contributing All 12 Months to Grids	
		*T*_*dmean*_	*VPD*_*min*_ *VPD*_*max*_	*T*_*dmean*_	*VPD*_*min*_ *VPD*_*max*_
ASOS	Hourly	1960	1947	1789	1619
RAWS	Hourly	1066	1067	885	682
COOP	Daily	388	0	334	0
OKMESONET	Hourly	129	129	123	122
SCAN	Hourly	116	116	87	76
AGRIMET	Hourly	73	73	70	55
NDBC	Hourly	72	72	53	56
COAGMET	Hourly	63	63	54	50
WBAN	Daily	42	0	38	0
Other Networks	Daily	23	0	23	0
Total Surface Stations		4001	3467	3456	2660
NCAR/NCEP (Upper-air) Grid Points	1981–2010 monthly mean	69^a^	69^a^	69^a^	69^a^

^a^ Count for upper air is the number of grid points used.

To improve marine representation, data were obtained from coastal stations and offshore buoys operated by the NOAA National Data Buoy Center (NDBC; http://www.ndbc.noaa.gov/data/historical/stdmet). To better define humidity profiles at high elevations, mean monthly upper-air temperature, geopotential height, and relative humidity grid points for the western and eastern United States were obtained at 2.5-degree resolution for the period 1981–2010 from the National Center for Environmental Prediction (NCEP) Global Reanalysis (ftp://ftp.cdc.noaa.gov/Datasets/ncep.reanalysis.derived/pressure). The 5650-m level (~500 hPa) was chosen for the western US, and the 3050-m level (~700 hPa) for the eastern US. (Upper-air data were not needed in the central US, because of a lack of elevated terrain.) These levels were sufficiently far above the highest terrain features to minimize potential errors involved in estimating surface humidity statistics from free air values.

### Quality Control and Calculation of Monthly Values

As shown in Table 1, station data were available at either an hourly or daily time step. Daily data were sufficient for *T*_*dmean*_, but hourly data were required to calculate *VPD*_*min*_ and *VPD*_*max*_. In some cases, hourly data were provided in the form of *RH* directly from the instrument. Range checks were performed to screen out values that were either impossible or could cause instabilities in the calculation of related statistics. If *RH* ≤ 0 or *RH* > 105, the value was set to missing. Otherwise, if *RH* < 0.5, it was set to 0.5, and if 100 < RH ≤ 105 (as sometimes occurs under saturated conditions), it was set to 100.

Dew point (*T*_*d*_) was derived from hourly *RH* (%) and ambient temperature (*T*_*a*,_°C) by calculating the saturation vapor pressure at *T*_*a*_ *(SATVP*_a_) ([26], Eq 21):

$$SATVP_{a}=6.1094e^{\left(\frac{17.625T_{a}}{243.04+T_{a}}\right)}$$ (1)

and finding *T*_*d*_ (°C) [27]:

$$T_{d}=\frac{237.3\text{ln}\left[SATVP_{a}\left(\frac{RH}{611}\right)\right]}{7.5\text{ln}10-\text{ln}\left[SATVP_{a}\left(\frac{RH}{611}\right)\right]}$$ (2)

A consistency check was done to ensure that the calculated *T*_*d*_ was less than *T*_a;_ if not, each was set to missing.

As a range check, *T*_*d*_ values that fell below -68°C, or exceeded the statewide extreme maximum temperature record (http://www.ncdc.noaa.gov/extremes/scec/records) were set to missing. In addition, the hourly *T*_*d*_ values were subjected to a step check in which each value could not differ from that of the previous hour by more than 10°C; otherwise the current hour’s *T*_*d*_ was set to missing.

Hourly *VPDs* were calculated from a combination of *T*_*a*_ and *T*_*d*_ or *T*_*a*_ and *RH*, depending on which was available. If *RH* was available, *VPD* was calculated by: (1) finding *SATVP*_*a*_ from Eq 1; (2) finding the saturation vapor pressure at the previously calculated *T*_*d*_:

$$SATVP_{d}=6.1094e^{\left(\frac{17.625T_{d}}{243.04+T_{d}}\right)}$$ (3)

and; (3) calculating *VPD* as

$$VPD=SATVP_{a}-SATVP_{d}$$ (4)

If *T*_*d*_ was available, *VPD* was obtained by calculating *SATVP*_*d*_ from Eq 3 and *VPD* from Eq 4. Each hourly *VPD* was subjected to a range check, where if *VPD* > 200 hPa or < 0 hPa, or if *VPD* ≥ *SATVP*_*a*_, the value was set to missing.

Three persistence tests were done on *T*_*a*_ and *T*_*d*_ hourly values for each 24-hour period. If the maximum difference between any pair of hourly observations or the difference between the maximum and minimum value over the 24-hour period was less than 0.1°C, or the standard deviation of all values in the 24-hour period was less than 0.1°C, the day was considered a “flat day” and all variables, including the *VPDs*, were set to missing.

For networks with hourly data, *T*_*dmean*_ values were calculated by averaging available hourly observations, subject to the requirement that at least 18 of 24 observations be non-missing; fewer than 18 non-missing hourly observations resulted in a missing daily value. Daily *VPD*_*min*_ and *VPD*_*max*_, as well as *T*_*min*_, and *T*_*max*,_ were determined by finding the minimum and maximum hourly value, respectively, subject to the same data completeness requirement.

Daily maximum and minimum *T*_*d*_ (*T*_*dmax*_, *T*_*dmin*_) and mean *T*_*a*_ (*T*_*mean*_) values were calculated for diagnostic purposes. Consistency checks were performed on daily combinations of *T*_*dmean*_ and temperature as follows: If *T*_*max*_ < *T*_*dmax*_, *T*_*min*_ < *T*_*dmin*_, or *T*_*mean*_ < *T*_*dmean*_, all variables, including *VPD*_*min*_ and *VPD*_*max*_, were set to missing.

The daily values were averaged to create monthly mean *T*_*dmean*_, *VPD*_*min*_, and *VPD*_*max*_ for each year of record. A minimum of 85% of non-missing daily values were required for a monthly value to be non-missing [28], [29].

The monthly averages were tested for spatial consistency using the ASSAY QC (quality control) system. ASSAY is a version of PRISM that estimates station values in their absence and compares them to the observed values, a procedure termed cross-validation [8]. The interpolation procedures in ASSAY are exactly the same as in PRISM; the only difference is that ASSAY interpolates to point (station) locations, rather than grid cells. As a rule, a large discrepancy between an observed station value and the interpolated estimate from ASSAY suggests that the station value is unusual compared to nearby stations, and may therefore be erroneous. In previous work, an ASSAY QC analysis was performed for *T*_*dmean*_, with *T*_*dmean*_ expressed in the form of dew point depression (*DPD*) with *T*_*min*_; that is, *DPD* = *T*_*min*_−*T*_*dmean*_. (As will be discussed in the next section, the *DPD* form of *T*_*dmean*_ was the favored method of expressing *T*_*dmean*_ for interpolation in this study.) In the previous ASSAY analysis of *DPD*, QC was done manually by trained personnel, and compared with the ASSAY results. It was found that when the absolute difference between the observation and ASSAY estimate of *DPD* exceeded 4.5°C, the value was considered erroneous by the manual method. Based on these results, in this study ASSAY QC was applied to *T*_*dmean*_ expressed as *DPD*, and station values producing absolute differences between the observation and estimate of more than 4.5°C were flagged as bad and set to missing. Overall, 0.8 percent of the monthly *T*_*dmean*_ values were set to missing using this method.

To take advantage of the ASSAY QC screening method, *VPD*_*min*_ and *VPD*_*max*_ were also expressed as *DPD* with *T*_*min*_, termed *DPD*(*VPD*_*min*_) and *DPD*(*VPD*_*max*_), respectively. This involved: (1) calculating the saturation vapor pressure for the station’s *T*_*min*_ value (*SATVP*_*a*_) using Eq 1; (2) subtracting the *VPD* from this value to get *SATVP*_*d*_; (3) obtaining *T*_*d*_ from Eq 2, substituting *SATVP*_*d*_ for *SATVP*_*a*_ and using 100 for the *RH* value; and (4) subtracting the resulting *T*_*d*_ from *T*_*min*_ to obtain the *DPD*. Manual QC checks found that the same threshold of 4.5°C used in QC’ing *DPD* was suitable for *DPD*(*VPD*_*min*_) and *DPD*(*VPD*_*max*_) as well, and those exceeding this threshold were flagged as bad and set to missing. Overall, 0.7 percent of the monthly *VPD*_*min*_ values and 1.8 percent of the monthly *VPD*_*max*_ values were set to missing using this method.

Monthly values of *T*_*dmean*_, *VPD*_*min*_, and *VPD*_*max*_ passing the ASSAY QC screening were averaged over their period of record (POR), or 1981–2010, if available. A 1981–2010 monthly mean calculated using data from at least 23 of these 30 years (75% data coverage) was considered to be sufficiently characteristic of the 1981–2010 period, and was termed a “long-term” station. However, many stations had a POR of fewer than 23 years. Averages from stations with short PORs were subjected to adjustment to minimize temporal biases, as described in [8], Appendix A.

## Mapping Methods

Mapping of 1981–2010 mean monthly *T*_*dmean*_, *VPD*_*min*_, and *VPD*_*max*_ was performed using PRISM [6–8], [30]. For each grid cell, PRISM calculated a local linear regression function between the atmospheric moisture variable and a predictor grid (see Climatologically-Aided Interpolation below). Nearby stations entering the regression were assigned weights based primarily on the physiographic similarity of the station to the grid cell. Physiographic factors relevant to this study were distance, elevation, coastal proximity, vertical atmospheric layer, and topographic position (relative to surrounding terrain). Detailed descriptions of the PRISM model algorithms, structure, input grids, and operation are given in [7], [8], and [30]. Details on the specific modeling approach for this study are given below.

### Derive or Interpolate VPDmin and VPDmax?

Given that *T*_*dmean*_ is a basic atmospheric moisture variable from which other variables can be derived, early in this study the question was asked: do *VPD*_*min*_ and *VPD*_*max*_ need to be interpolated separately, or can they be derived from a combination of the *T*_*dmean*_ grid and previously-created grids of *T*_*max*_ and *T*_*min*_? More specifically, can *T*_*dmean*_ be combined with *T*_*max*_ to estimate *VPD*_*max*_ and *T*_*dmean*_ combined with *T*_*min*_ to estimate *VPD*_*min*_? To do this, we must assume that *T*_*d*_ = *T*_*dmean*_ and *T*_*a*_ = *T*_*max*_ at the time of *VPD*_*max*_, and *T*_*d*_ = *T*_*dmean*_ and *T*_*a*_ = *T*_*min*_ at the time of *VPD*_*min*_. To test these assumptions, monthly averages of *VPD*_*min*_ and *VPD*_*max*_, as well as *VPD*_*min*_ and *VPD*_*max*_ estimated as described above, were calculated and averaged for each month over the ten-year period 2003–2012 at 100 randomly selected ASOS stations with hourly data. Results of this exercise are given in Table 2 for January and July, which bracket the range of monthly values observed during the year.

**Table 2:** Monthly averages of actual and estimated vapor pressure deficit. 2003–2012 January and July averages from 100 randomly-selected ASOS stations. Actual and estimated *VPD* values are expressed as 5^th^ percentile / mean / 95^th^ percentile.

*VPD* (hPa)	January	July
***VPD***_***min***_	0.2 / 0.8 / 1.6	0.9 / 3.3 / 9.7
***Estimated VPD***_***min***_	-0.6 / 0.7 / 1.6	-0.1 / 4.2 / 11.2
***VPD***_***max***_	1.5 / 5.9 / 12.3	15.2 / 26.9 / 47.7
***Estimated VPD***_***max***_	1.8 / 6.3 / 12.1	14.7 / 26.3 / 45.4

In Table 2, actual and estimated *VPD*_*min*_ and *VPD*_*max*_ are expressed as means and percentiles (5^th^ percentile / mean / 95^th^ percentile) so that the full distribution of values can be evaluated. Differences between actual and estimated *VPD*_*max*_ were generally within about three hPa or five percent across the distributions in both winter and summer. However, a proportion of the estimated *VPD*_*min*_ values fell below zero, which is not physically possible. This problem was most serious during winter; in January, a *VPD*_*min*_ of less than zero was estimated at 47 of the 100 stations. In these cases, *T*_*dmean*_ exceeded *T*_*min*_, resulting in a negative *VPD*. In reality, the opposite was true, resulting in a positive *VPD*_*min*_. Given this issue, deriving *VPD*_*min*_ and *VPD*_*max*_ from *T*_*dmean*,_ *T*_*min*,_ and *T*_*max*_ was not considered a viable option, leading us to interpolate *VPD*_*min*_ and *VPD*_*max*_ directly from station data.

### Climatologically-Aided Interpolation

In previous work, constructing the 1971–2000 and 1981–2010 monthly normals for *T*_*min*_ and *T*_*max*_ used a DEM as the predictor grid (see [8] for details on methods used for mapping *T*_*min*_ and *T*_*max*_). There were nearly 10,000 stations used in the mapping of the 1971–2000 *T*_*min*_ and *T*_*max*_ normals, and over 16,000 stations used in the 1981–2010 normals. In contrast, there were only 3,500–4,000 stations available for this study, with poor representation at high elevations. Faced with limited station data, we opted for the CAI method of interpolation. CAI uses an existing climate grid to improve the interpolation of another climate element for which data may be sparse or intermittent in time [31–36]. This method relies on the assumption that local spatial patterns of the climate element being interpolated closely resemble those of the existing climate grid (called the predictor grid). Uses of CAI fall into two main categories: (1) using a long-term mean grid of a climate element to aid the interpolation of the same element over different (usually shorter) averaging periods; and (2) using a grid of a climate variable to aid the interpolation of a different, but related, climate variable, such as interpolating annual extreme minimum temperature using January mean minimum temperature as the predictor grid [37]. A classic example of the first strategy involves mapping a long-term mean climatology carefully with sophisticated methods, then developing time series grids for shorter averaging periods (monthly or daily) using simpler and faster methods such as inverse-distance weighting to interpolate deviations from the mean climatology to a grid. These deviations can then be added to (e.g., temperature) or multiplied by (e.g., precipitation) the mean climatology to obtain the new grid.

**Fig 4:**
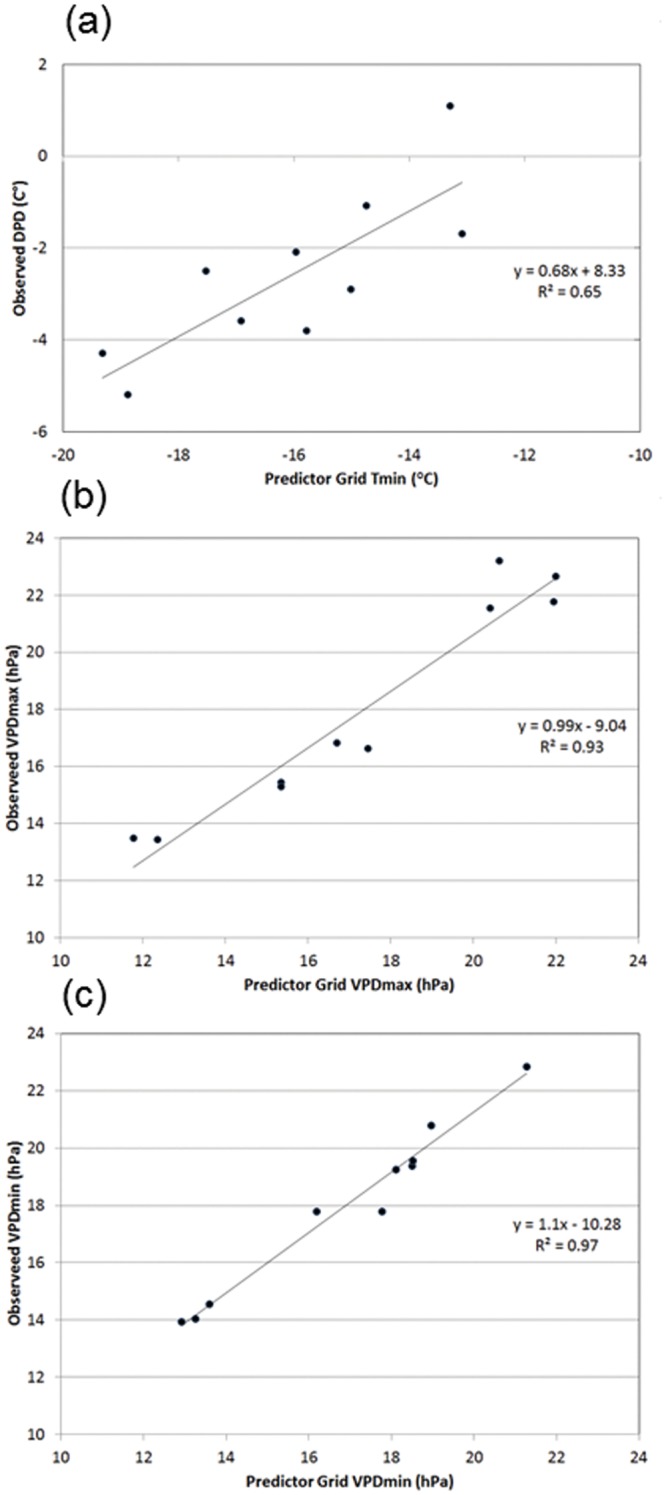
Scatterplots of relationships between predictor grid and interpolated variables. 1981–2010 mean monthly (a) dew point depression (*DPD)* vs. minimum temperature (*T*_*min*_) for January in western Wyoming; (b) minimum vapor pressure deficit (*VPD*_*min*_) vs. first-guess *VPD*_*min*_ for June near Las Vegas; and (c) maximum vapor pressure deficit (*VPD*_*max*_) vs. first-guess *VPD*_*max*_ for October in San Francisco.

Our use of CAI for this study falls into the second strategy, for which we use pre-existing grids of 1981–2010 mean monthly *T*_*min*_ and *T*_*max*_ as predictor grids in the interpolation process. A series of tests was conducted with ASSAY and PRISM to determine which of the 1981–2010 mean monthly *T*_*min*_ and *T*_*max*_ grids were the strongest predictors of the spatial patterns of *T*_*dmean*_. *T*_*min*_ was found to be a good predictor, which is not surprising given that temperatures often reach the dew point at the time of *T*_*min*_ over much of the US. However, inspection of the interpolated grids revealed that in some areas subject to locally low temperatures, such as cold air pools in mountain valleys, the interpolated *T*_*dmean*_ exceeded the mean temperature, meaning that long-term mean *RH* exceeded 100 percent, which was not acceptable. In order to more closely tie the patterns of *T*_*dmean*_ to the patterns of the existing monthly *T*_*min*_ grids and their relatively large supporting station data sets, each *T*_*dmean*_ station value was expressed as the deviation from *T*_*min*_, or *DPD* (*T*_*dmean—*_ *T*_*min*_). Thus, in the interpolation process, PRISM was run with the 1981–2010 mean monthly *T*_*min*_ grid as the independent variable and *DPD* as the dependent variable. The local regression functions were generally not as strong as they were in the case of *T*_*dmean*_ vs. *T*_*min*_, because *DPD* was essentially the residual from the *T*_*dmean*_ vs. *T*_*min*_ relationship. Once the mean monthly *DPD* values were mapped in this manner, the final *T*_*dmean*_ grid was obtained by adding the *DPD* grid to the 1981–2010 *T*_*min*_ grid. The result was an interpolated *T*_*dmean*_ grid that was highly consistent in an absolute, as well as relative sense, with the associated *T*_*min*_ grid. An example of the relationship between 1981–2010 January mean observed *DPD* and gridded *T*_*min*_ for a location north of the Wind River Mountains of Wyoming (43.6N, 109.73W) is shown in Fig 4a. This area is characterized by persistent wintertime cold air pools in mountain valleys, where the humidity is high and *T*_*min*_ is less than *T*_*dmean*_ (*DPD*<0). Above these cold pools, *T*_*min*_ increases and rises above *T*_*dmean*_ (*DPD*>0).

An additional series of tests was conducted with PRISM and ASSAY to determine whether either of the pre-existing 1981–2010 mean monthly *T*_*min*_ and *T*_*max*_ grids could be used as predictors of the spatial patterns of *VPD*_*min*_ and *VPD*_*max*_. *T*_*max*_ was found to be a good predictor of *VPD*_*max;*_ and the same was true for *T*_*min*_ and *VPD*_*min*_. However, once *T*_*dmean*_ was mapped, a superior, second-generation CAI method using *T*_*dmean*_ became available. Termed CAI2, this method involves using the result of a CAI interpolation as the predictor grid in another CAI interpolation. Specifically, a first-guess *VPD*_*min*_ grid was created by calculating the *VPD* associated with the combination of the *T*_*dmean*_ and *T*_*min*_ grids using Eqs 1, 3 and 4. Similarly, a first-guess *VPD*_*max*_ grid was found by calculating the *VPD* associated with the combination of the *T*_*dmean*_ and *T*_*max*_ grids. These “first-guess” predictor grids represented what the *VPD*_*min*_ and *VPD*_*max*_ spatial patterns would be like if *T*_*d*_ was held constant throughout the day at *T*_*dmean*_, and *VPD*_*min*_ occurred at the time of *T*_*min*_ and *VPD*_*max*_ occurred at the time of *T*_*max*_. While these assumptions are not always correct (see Table 2), they are sufficiently reasonable to produce predictor grids that closely match the relative spatial patterns of *VPD*_*min*_ and *VPD*_*max*_.

An example of the relationship between 1981–2010 June mean observed *VPD*_*min*_ and first-guess predictor grid *VPD*_*min*_ for a location near Las Vegas, Nevada (35.79N, 115.26W) is shown in Fig 4b. In this desert environment, *VPD*_*min*_ is still relatively large (14–22 hPa), even at its minimum for an average day in June. The lowest *VPD*_*min*_ values are found at higher elevations, where temperatures are cooler. An example of the relationship between 1981–2010 October mean observed *VPD*_*max*_ and the first-guess predictor grid *VPD*_*max*_ for a location in San Francisco, along the California coastline (37.76N, 122.45W), is shown in Fig 4c. In this case, the lowest *VPD*_*max*_ values are found along the immediate coast where temperatures are cooler and moisture is greater, with higher values in warmer and drier inland areas.

### PRISM Weighting Functions

During PRISM interpolation, upon entering the local linear regression function for a pixel, each station was assigned a weight based on several factors. The combined weight (*W*) of a station is given by the following:

$$W=W_{c}{\left[F_{d}W_{d}{}^{2}+F_{z}W_{z}{}^{2}\;\right]}^{1/2}W_{p}W_{l}W_{t}$$ (5)

where *W*_*c*_, *W*_*d*_, *W*_*z*_, *W*_*p*_, *W*_*l*_, and *W*_*t*_ are cluster, distance, elevation, coastal proximity, vertical layer, and topographic position weights, respectively, and *F*_*d*_ and *F*_*z*_ are user-specified distance and elevation weighting importance scalars [8], [38]. All weights and importance factors, individually and combined, are normalized to sum to unity. PRISM weighting functions not enabled in this study were topographic facet weighting, which is used primarily to identify rain shadows in precipitation mapping, and effective terrain height weighting, which identifies orographic precipitation regimes based on terrain profiles [8].

Table 3 summarizes how the PRISM climate regression and station weighting functions accounted for physiographic climate forcing factors, and provides citations for further information. Cluster weighting was used to keep clusters of stations that represent similar local conditions from dominating the regression functions; both horizontal and vertical separations were considered [8]. Distance and elevation weighting were used to accommodate the spatial coherence of climatic regimes, both horizontally and vertically [8].

**Table 3:** PRISM weighting algorithms and associated physiographic climate forcing factors. Inputs to the algorithms and references on their formulation and use are included. Methods used to prepare the gridded model inputs are summarized in [8], Tables 1 and 2.

PRISM Algorithm	Description	Physiographic Forcing Factors	Inputs to Algorithm	Reference
**Climate Regression Function (CAI)**	Develops local relationships between a climate predictor grid and the interpolated variable	Incorporates physiographic features implicit in the climate predictor grid	Station data; climate predictor grid	This paper, Mapping Methods Section; [37] Section 4b
**Cluster Weighting**	Downweights stations clustered with others	--	Station locations and elevations	[8], Appendix B
**Distance Weighting**	Upweights stations that are horizontally close	Horizontal coherence of climate regimes	Station locations	[8], Section 4.2.1
**Elevation Weighting**	Upweights stations that are vertically close	Vertical coherence of climate regimes	DEM; station locations and elevations	[7], Section 4.1
**Coastal Proximity Weighting**	Upweights stations having similar exposure to coastal influences	Effects of water bodies on temperature and moisture	DEM; coastal proximity grid; station locations	[7], Section 6; [30] Section 2.3.2; [8] Section 4.2.2
**Two-Layer Atmosphere Weighting**	If an inversion is present, upweights stations in the same vertical layer (boundary layer or free atmosphere)	Temperature inversions; vertical limit to moist boundary layer (humidity)	DEM; inversion height grid; station locations	[7], Section 7; [30], Section 2.3.4
**Topographic Position Weighting**	Upweights stations having similar heights above the local terrain	Cold air drainage and pooling in topographic depressions	DEM; topographic index grid; station locations	[38], Section 4; [8], Section 4.2.5

Coastal proximity weighting accounted for sharp gradients in temperature and atmospheric moisture from coastlines to interior regions [7], [8], [30]. Atmospheric layer weighting was useful where temperature inversions occurred, by delineating gradients in the relationship between temperature and atmospheric moisture along the transition from the relatively humid boundary layer near the earth’s surface to the drier free atmosphere above [7], [30]. Topographic position weighting differentiated topographically sheltered locations where cold, moist air may accumulate, from more exposed locations not susceptible to cold air pooling, such as hill slopes and ridge tops [8], [38].

Relevant PRISM control parameters are listed in Table 4. A minimum of 25 stations were required for each pixel’s regression function, and the radius of influence was expanded from a minimum of 20 km to as far as necessary to reach the 25-station threshold. *T*_*dmean*_ slope bounds were expressed as the change in *DPD* per unit *T*_*min*_ from the predictor grid, and *VPD* slopes expressed as the change in *VPD* per unit first-guess *VPD* from the predictor grid. The maximum and minimum allowable regression slopes were derived from test runs of PRISM where distributions of slope values were created and outliers examined to determine validity, combined with performance assessments using ASSAY. In the final PRISM interpolation runs, slopes falling outside the designated bounds were set to values that fell halfway between the default slope and the bound that the slope violated (either the maximum or minimum). In the western region, allowable slopes in the relationship between *VPD*_*max*_ and the first-guess *VPD*_*max*_ were constrained to fall between 0.99 and 1.01, so as to avoid a rare circumstance of predicting a *VPD*_*max*_ that might approach the value of *SATVP*_a_ at *T*_*max*_ (hence producing a very low *RH*) in the warmest and driest areas. The exponential relationship between temperature and *SATVP*_a_ steepens at higher temperatures, leaving less room for error in extremely low-humidity situations. Unconstrained, the average slope of the 5.5 million PRISM regression functions (one per pixel) for July (the warmest month) across the western region was 1.017, with the 10th percentile at 0.964 and the 90th at 1.15, so this constraint had a very slight effect.

**Table 4:** Values of relevant PRISM interpolation parameters. Regression slope bounds are sometimes different among regions; values separated by forward slashes are for west, central, and east regions, respectively. See [7] and [8] for details on PRISM parameters and Table 3 for citations to details on weighting function formulations.

Name	Description	*T*_*dmean*_	*VPD*_*min*_	*VPD*_*max*_
Regression Function				
*R*	Radius of influence	20 km*	20 km*	20 km*
*s*_*t*_	Minimum number of total stations desired in regression	25 stations	25 stations	25 stations
*β*_*1ma*_	Min valid regression slope, layer 1	-0.5^+^	-10/-1/-1.5^#^	0.99/0/0^#^
*β*_*1xa*_	Max valid regression slope, layer 1	2.5^+^	10/2/1^#^	1.01/1.5/1.5
*β*_*1da*_	Default regression slope, layer 1	0.5^+^	1/0.5/0.5^#^	1/1/1^#^
*β*_*1mb*_	Min valid regression slope, layer 2	-0.5^+^	-10/-1/-1^#^	0.99/0/0^#^
*β*_*1xb*_	Max valid regression slope, layer 2	2.5^+^	10/2/1^#^	1.01/1.5/1.5^#^
*β*_*1db*_	Default regression slope, layer 2	0.5^+^	1/1/0.5^#^	1/1/1^#^
Distance Weighting				
*A*	Weighting exponent	1.5	1.5	1.5
*F*_*d*_	Importance factor	0.5	0.5	0.5
*D*_*m*_	Minimum allowable distance	0 km	0 km	0 km
Elevation Weighting				
*B*	Weighting exponent	1.5	1.5	0.5
*F*_*z*_	Importance factor	0.5	0.5	0.5
*Δz*_*m*_	Station-grid cell elev difference below which weight is maximum	100 m	100 m	100 m
*Δz*_*x*_	Station-grid cell elev difference above which weight is zero	5000 m	5000 m	5000 m
Coastal Proximity Weighting				
*v*	Weighting exponent	1.0	1.0	1.0
*Δp*_*m*_	Station-grid cell proximity difference below which weight is maximum	100^$^	100^$^	100^$^
*Δp*_*x*_	Station-grid cell proximity difference above which weight is zero	300^$^	300^$^	300^$^
Vertical Layer Weighting				
*y*	Weighting exponent	0.5	0.5	0.5
*Δl*_*m*_	Station-grid cell distance to adjacent layer below which weight is maximum	100 m	100 m	100 m
*Δl*_*x*_	Station-grid cell distance to adjacent layer above which weight is zero	N/A	N/A	N/A
Topographic Position Weighting				
*μ*	Weighting exponent	1.5	1.5	1.5
*Δt*_*m*_	Station-grid cell topo difference below which weight is maximum	50 m	50 m	50 m
*Δt*_*x*_	Station-grid cell topo difference above which weight is zero	900 m	900 m	900 m

* Expands as needed to encompass minimum number of total stations desired in regression (*s*_*t*_).

^+^ Slope is expressed as the change in dew point depression (*DPD*) per unit Tmin from the predictor grid

^#^ Slope is expressed as the change in *VPD* per unit first-guess *VPD* from the predictor grid

^$^ Units are effective distance in grid cells from the coast, incorporating terrain barriers

Station weighting parameters were very similar among the three variables, and were generally set to the same values across the three regions. The exception was the elevation weighting exponent for *VPD*_*max*_, which was set to 0.5, compared to 1.5 for the other variables. *T*_*max*_ and *VPD*_*max*_ occur primarily during the day, when the atmosphere is more likely to be well-mixed, and vertical gradients in atmospheric moisture more slowly varying. As a rule, the weighting exponents were set to the lowest values possible to achieve the desired effects. Being parsimonious with the weighting exponents ensured that the station data entering the local regression functions were not down-weighted unnecessarily, which can weaken the statistical results by decreasing the effective number of stations in the regression.

A number of small but noticeable inconsistencies in *DPD* values between adjacent stations were observed during the summer months in some agricultural areas, most notably in eastern Colorado. Further investigation revealed that relatively low *DPD* values were coming from two networks: AGRIMET and COAGMET. Stations in these networks are typically sited in or near irrigated fields for use in water management calculations, resulting in more humid conditions than locations away from irrigated areas. Given that the data from these networks were of otherwise high quality, it was unreasonable to simply omit these two networks outright. This issue raised questions about whether the grids should, or could, represent conditions over irrigated land. Concerns over attempting to do so are summarized as follows:

1. Siting requirements for these station networks state that the station must be located in, or immediately adjacent to, an irrigated field to be representative of an irrigated environment. This suggests that the effects of irrigation on humidity are highly local, and which may not extend beyond one 800-pixel. In practice, however, these stations influenced the interpolated estimates many km away, well beyond the likely limits of irrigation in many instances.
2. A complete picture of the location and extent of irrigated lands across the country was unavailable; further, there was no interpolation mechanism in place to constrain the influence of stations on irrigated fields to just those lands. It was also unclear how to modify *DPD* values in the many irrigated areas not represented by station data.
3. We, and likely others, will be using these atmospheric moisture climatologies as the predictor grids for CAI mapping of monthly and daily time series of the same variables. Given that many of these time series will extend back a century or more, when crop patterns and irrigation practices were very different than today, patterns of humidity caused by today’s irrigation patterns could be propagated to times when they are not applicable.

Given these concerns, a subjective middle ground was taken, where a few (<10) stations causing the most severe spatial discrepancies were omitted from the *T*_*dmean*_ dataset, and the rest retained. For consistency, the same stations were also omitted from the *VPD*_*min*_ and *VPD*_*max*_ datasets.

### Grid Post-processing

Once monthly grids of *T*_*dmean*_, *VPD*_*min*_, and *VPD*_*max*_ were generated with PRISM, post-processing checks were made to ensure that the interpolated values did not exceed reasonable ranges, and that consistency was maintained among the three variables and with the pre-existing 1981–2010 mean monthly *T*_*min*_ and *T*_*max*_ grids. Given that the 1981–2010 monthly means are by definition made up of yearly values above and below these means, the acceptable ranges were defined to be more restrictive than what would be allowed on, say, a single day. If *T*_*dmean*_ > (*T*_*mean*_− 0.5°C), *T*_*dmean*_ mean was set equal to *T*_*mean*_− 0.5°C. If *VPD*_*min*_ < 0.01 hPa, *VPD*_*min*_ was set equal to 0.01 hPa. If *VPD*_*min*_ < 0.001 *SATVP*_a_ at *T*_*min*_ (i.e., *RH* > 99.9%), *VPD*_*min*_ was set equal to 0.001 *SATVP*_a_ at *T*_*min*_. If *VPD*_*max*_ > 0.95 *SATVP*_a_ at *T*_*max*_ (i.e., *RH* < 5%), *VPD*_*max*_ was set equal to 0.95 *SATVP*_a_ at *T*_*max*_. The number of grid cells affected by these restrictions was limited to a handful in remote mountainous regions of the western US.

## Results and Discussion

### Spatial Patterns

#### Dew Point

**Fig 5:**
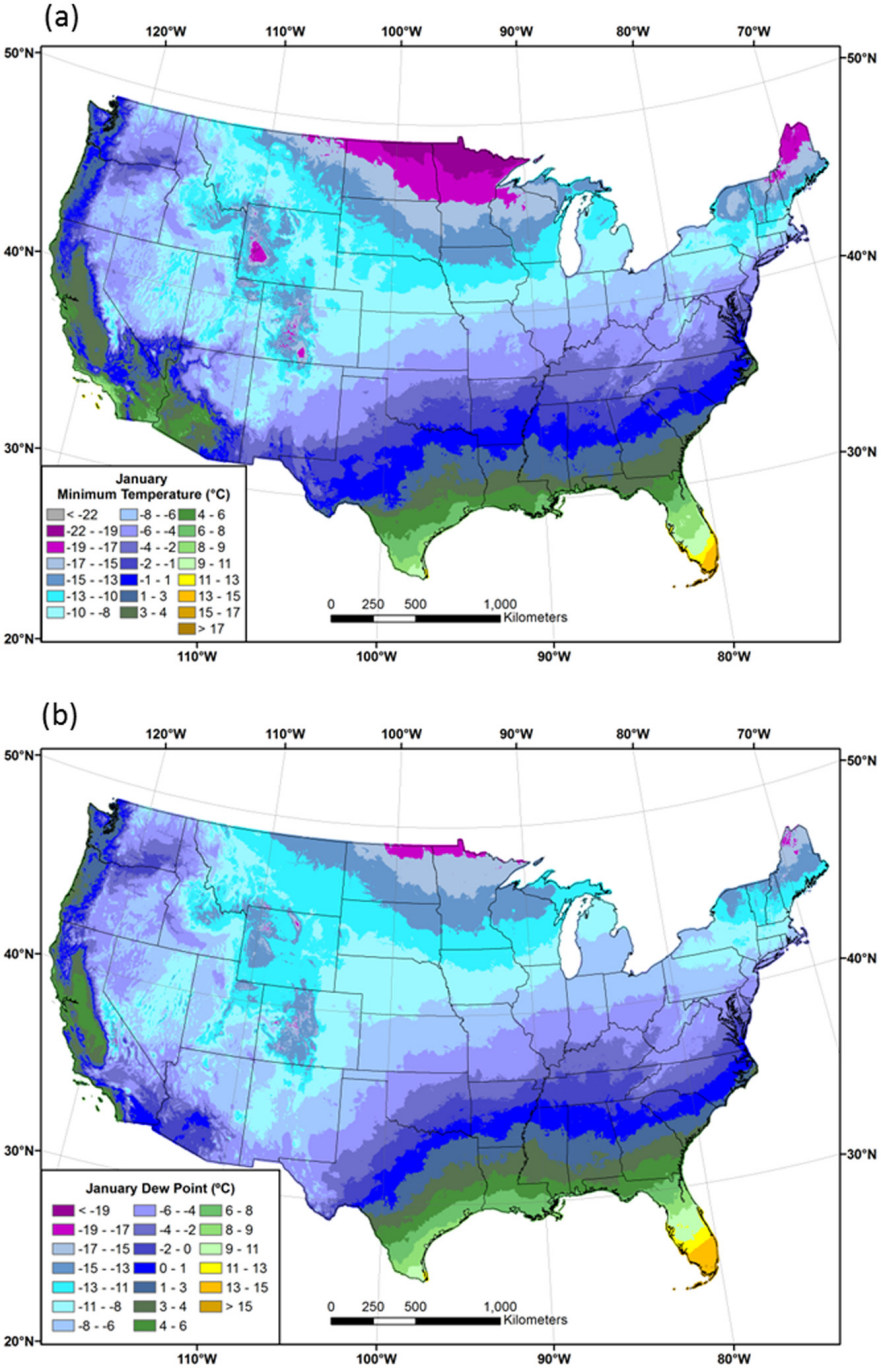
Maps of January minimum temperature and dew point. Conterminous US 1981–2010 mean January (a) minimum temperature (*T*_*min*_) and (b) dew point temperature (*T*_*dmean*_).

**Fig 6:**
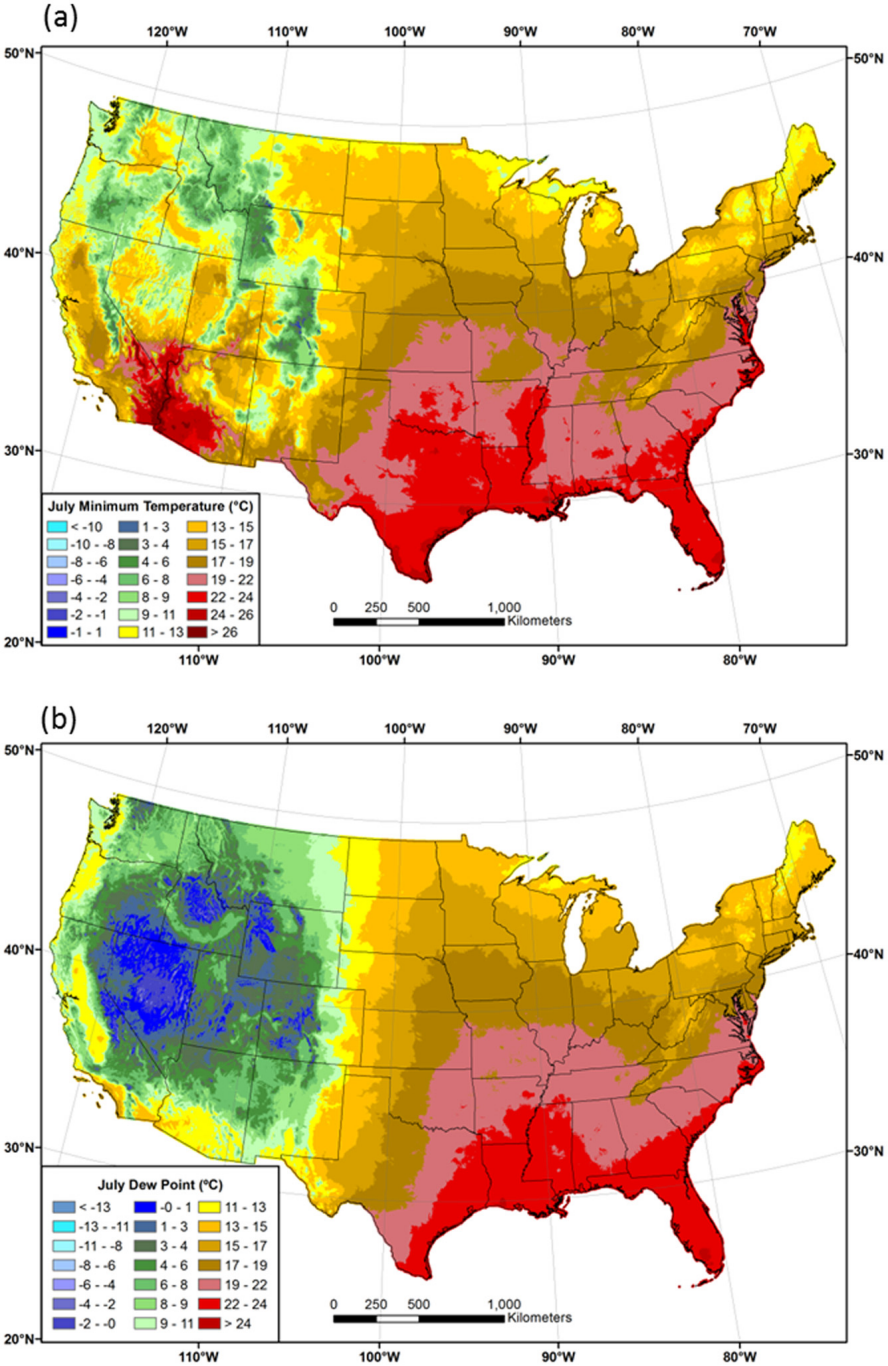
Maps of July minimum temperature and dew point. Conterminous US 1981–2010 mean July (a) minimum temperature (*T*_*min*_) and (b) dew point temperature (*T*_*dmean*_).

Maps of 1981–2010 mean *T*_*min*_ and *T*_*dmean*_ in January and July are shown in Figs 5 and 6, respectively. The general patterns of *T*_*dmean*_ are similar to those of *T*_*min*_, especially in winter. In January, values are lowest in the northern tier of states, and in cold pools along valley bottoms in the West (Fig 5). *T*_*dmean*_ is high in the eastern part of the country in July, exceeding 20°C in much of the Southeast, which is exposed to the transport of moist air from the Gulf of Mexico (Fig 6). Despite relatively warm temperatures, July *T*_*dmean*_ is low in the dry Intermountain West. A notable exception is the Southwest, where the summer monsoon produces locally elevated *T*_*dmean*_ values.

**Fig 7:**
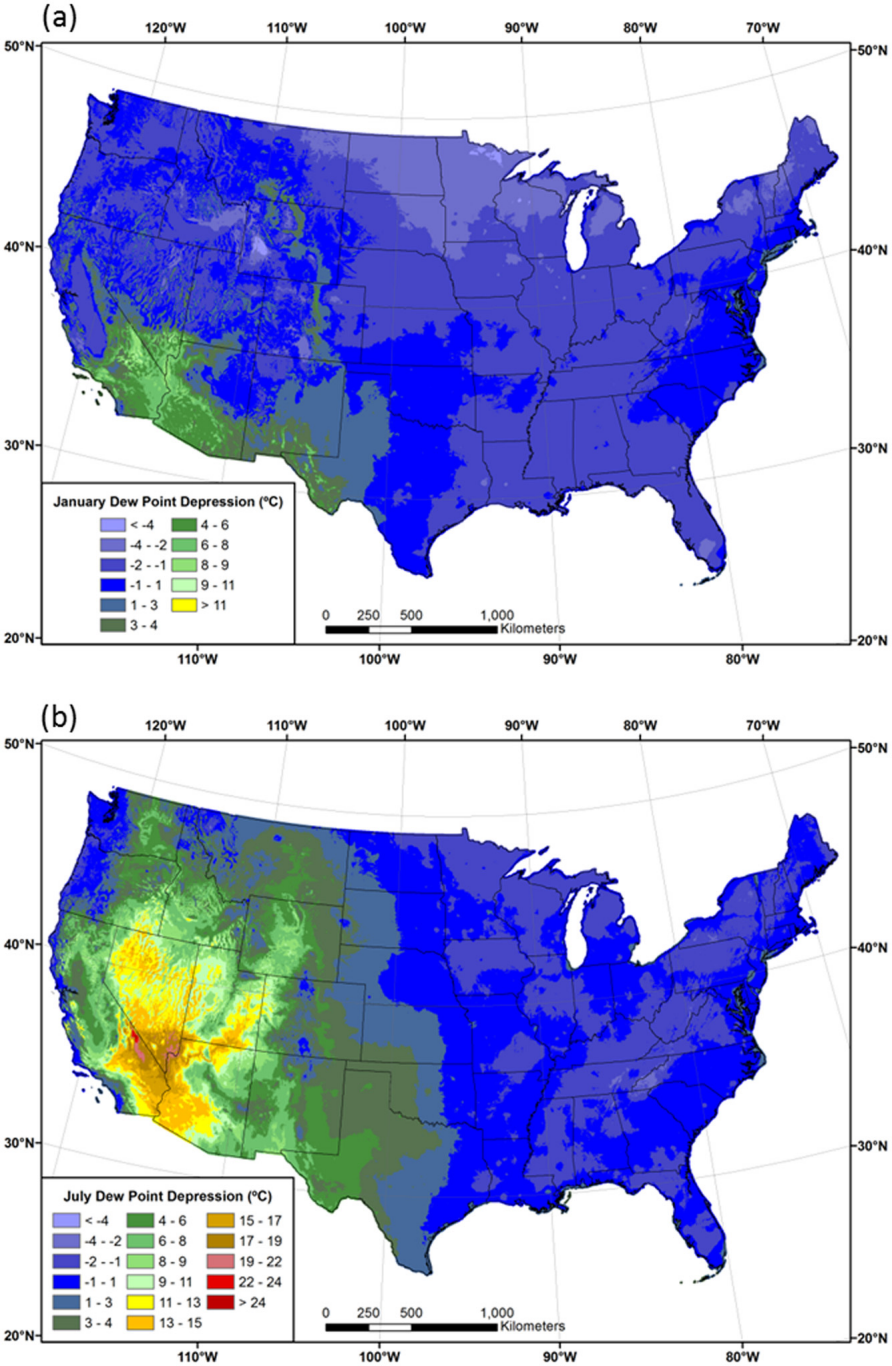
Maps of dew point depression *(DPD)*. Conterminous US difference between the 1981–2010 mean minimum temperature and the 1981–2010 mean dew point temperature (*T*_*min*_—*T*_*dmean*_) for (a) January and (b) July.

Interpolated *DPDs* between *T*_*min*_ and *T*_*dmean*_ (*T*_*min*_—*T*_*dmean*_) for January and July are shown in Fig 7. *DPD* is the original variable interpolated with PRISM before being added to the *T*_*min*_ grid to obtain *T*_*dmean*_. In January, *DPDs* are mostly near zero or negative, meaning that *T*_*dmean*_ is similar to, or greater than, *T*_*min*_ over much of the country. In the northern US and in persistent cold pools in large inland valleys of the West, *T*_*dmean*_ is as much as 3–5°C greater than *T*_*min*_ (Fig 7a). For example, the 1981–2010 mean January *T*_*dmean*_ at the International Falls, MN ASOS station is -18.1°C and the mean January *T*_*min*_ is -21.3°C. In contrast, *T*_*dmean*_ averages 6–8°C lower than *T*_*min*_ in the southwestern US, which is a testament to the region’s aridity, even in winter.

In July, *T*_*dmean*_ and *T*_*min*_ have similar values in the eastern half of the country. Interestingly, major metropolitan areas such as St. Louis, Kansas City, Minneapolis, and Detroit, and several eastern seaboard cities, are visible as small areas of relatively warm *T*_*min*_ values, resulting in positive differences between *T*_*min*_ and *T*_*dmean*_ of 1–3°C (dark green spots in Fig 7b). The West is dominated by larger positive differences, where *T*_*min*_ is much higher than *T*_*dmean*_. The changeover from near-zero to positive differences roughly follows the 100^th^ meridian. Notable exceptions are the West Coast, where cool, marine air penetrates inland, and areas of the Southwest affected by the summer monsoon. The core area of maximum *DPD* is centered on southeastern California, Nevada, western Arizona, and lower elevations of Utah, where *T*_*dmean*_ averages 10–25°C lower than *T*_*min*_. A combination of low moisture content (low *T*_*dmean*_) and limited time for nighttime cooling (high *T*_*min*_) during summer contribute to these high *DPDs*. Methods that estimate *T*_*dmean*_ by assuming it is equal to *T*_*min*_ would experience the largest errors in this region [22].

#### Vapor Pressure Deficit

**Fig 8:**
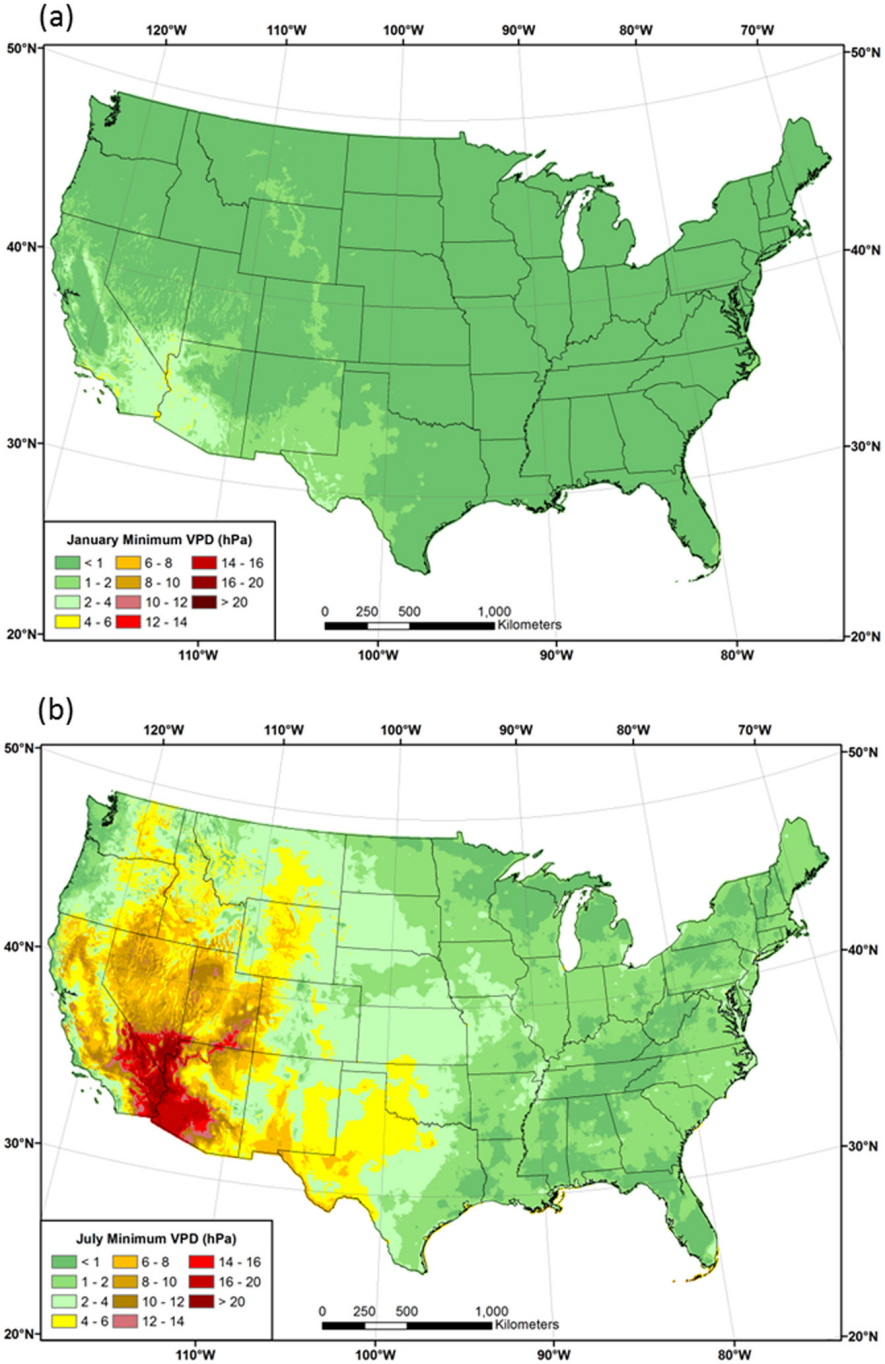
Maps of mean minimum vapor pressure deficit (*VPD*_*min*_). 1981–2010 mean monthly *VPD*_*min*_ for (a) January and (b) July.

Patterns of 1981–2010 mean *VPD*_*min*_ in January and July are shown in Fig 8. In winter, *VPD*_*min*_ is low (<1 hPa) over most of the country, due to a combination of low temperatures and relatively small differences between *T*_*a*_ and *T*_*d*_ during the morning hours (Fig 8a). Vapor pressure varies exponentially with temperature; for example, a difference between *T*_*a*_ and *T*_*d*_ of 1°C amounts to a *VPD* of 0.15 hPa at 20°C, but less than 0.05 hPa at 0°C. In July, *VPD*_*min*_ is also low in the eastern US, but exceeds 5 hPa over much of the West, reaching maxima of over 20 hPa in the desert southwest (Fig 8b).

**Fig 9:**
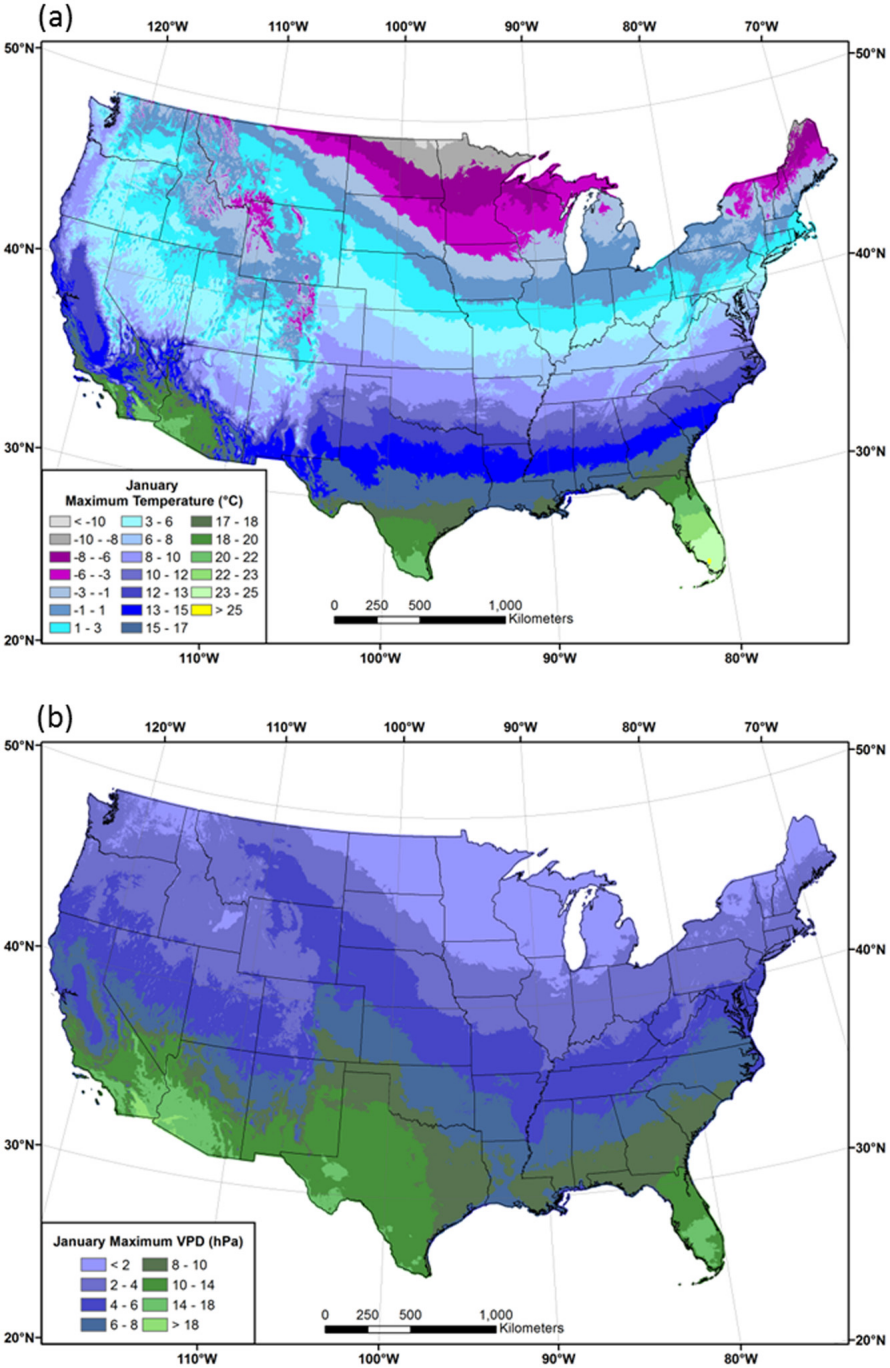
Maps of January maximum temperature and vapor pressure deficit. Conterminous US 1981–2010 mean January (a) maximum temperature (*T*_*max*_) and (b) maximum vapor pressure deficit (*VPD*_*max*_).

**Fig 10:**
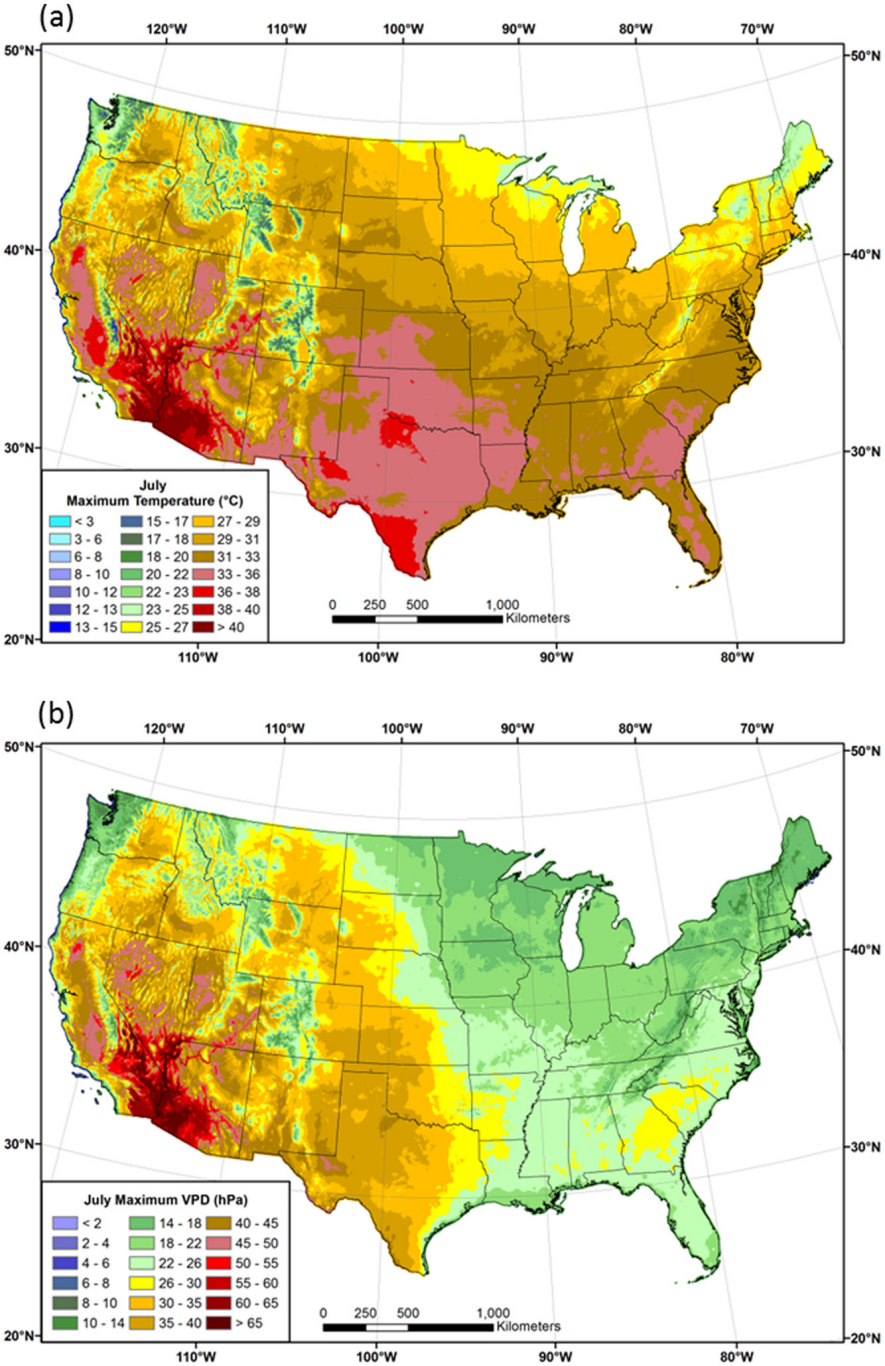
Maps of July maximum temperature and vapor pressure deficit. Conterminous US 1981–2010 mean July (a) maximum temperature (*T*_*max*_) and (b) maximum vapor pressure deficit (*VPD*_*max*_).

Spatial patterns of 1981–2010 mean *T*_*max*_ and *VPD*_*max*_ in January and July are shown in Figs 9 and 10, respectively. Patterns of *VPD*_*max*_ roughly follow those of *T*_*max*_ in January, with the lowest values in the northern tier and western mountains, and the highest in the southern states (Fig 9). In July, the area of high *VPD*_*max*_ expands considerably, exceeding 25 hPa over much of the western US, and reaching a maximum of over 60 hPa in the desert southwest (Fig 10). Coastal regions of the West exhibit relatively low *VPD*_*max*_ values, as they did for *DPD*. Similarly low values are found at higher elevations, illustrating the strong spatial relationship between *VPD*_*max*_ and *T*_*max*_. In the east, *VPD*_*max*_ values in the Midwest and northeast are mostly less than 20 hPa, while those in the southeast can range above 25 hPa; a notable maximum occurs in the piedmont region of Georgia and the Carolinas.

#### Relative Humidity Derivation

*RH* can be derived from *VPD* and *T*_*a*_ by calculating *SATVP*_a_ at the desired temperature from Eq 1, and factoring in the appropriate *VPD*:

$$RH=100(SATVP_{a}-VPD)/SATVP_{a}$$ (6)

On a gridded basis, if only *T*_*max*_ and *T*_*min*_ are available to estimate *T*_*a*_, and only *VPD*_*min*_ and *VPD*_*max*_ available to estimate *VPD*, grids of minimum *RH* (*RH*_*min*_) can be approximated by substituting *VPD*_*max*_ for *VPD* and *T*_*max*_ for *T*_*a*_, and maximum *RH* (*RH*_*max*_) can be similarly derived using *VPD*_*min*_ and *T*_*min*_. In performing these derivations, we make the assumption that *T*_*a*_ = *T*_*min*_ and *VPD* = *VPD*_*min*_ at the time of day when *RH*_*max*_ occurs, and *T*_*a*_ = *T*_*max*_ and *VPD* = *VPD*_*max*_ at the time of day when *RH*_*min*_ occurs. To evaluate the error introduced by these assumptions, we calculated 2003–2012 mean monthly *RH*_*min*_ and *RH*_*max*_ for the same 100 ASOS stations used in Table 2 and compared them with the estimated *RH*_*min*_ that would result from substituting *VPD*_*max*_ for *VPD* and *T*_*max*_ for *T*_*a*_, and *RH*_*max*_ resulting from using *VPD*_*min*_ and *T*_*min*_. As expected, *T*_*max*_ and *VPD*_*max*_ were slightly higher than *T*_*a*_ and *VPD* at the hour of *RH*_*max*_, and *T*_*min*_ and *VPD*_*min*_ were slightly lower than *T*_*a*_ and *VPD* at the hour of *RH*_*min*_ (Table 5). As a result, the substitutions resulted in an overestimation of *RH*_*min*_ and an underestimation of *RH*_*max*_. The differences averaged less than five percent in all months, suggesting that the derived grids are reasonable, but not exact, measures of the true *RH*.

**Table 5:** Results of temperature and vapor pressure deficit substitution in the derivation of minimum and maximum relative humidity. 2003–2012 January and July averages from 100 randomly-selected ASOS stations. Actual and estimated *RH* values are expressed as a distribution: 5^th^ percentile / mean / 95^th^ percentile.

Variable	January	July
***T***_***a***_ **at the hour of *RH***_***min***_ **(°C)**	4.7	29.8
***T***_***max***_ **(°C)**	6.2	30.2
***VPD* at the hour of *RH***_***min***_ **(hPa)**	5.7	26.4
***VPD***_***max***_ **(hPa)**	5.9	26.9
***RH***_***min***_ **(%)**	19 / 44 / 64	14 / 39 / 55
***Estimated RH***_***min***_ **(%)**	20 / 48 / 67	15 / 40 / 55
***T***_***a***_ **at the hour of *RH***_***max***_ **(°C)**	-1.1	19.2
***T***_***min***_ **(°C)**	-4.2	18.4
***VPD* at the hour of *RH***_***max***_ **(hPa)**	0.8	3.4
***VPD***_***min***_ **(hPa)**	0.8	3.3
***RH***_***max***_ **(%)**	51 / 86 / 94	51 / 85 / 96
***Estimated RH***_***max***_ **(%)**	50 / 85 / 93	50 / 85 / 96

**Fig 11:**
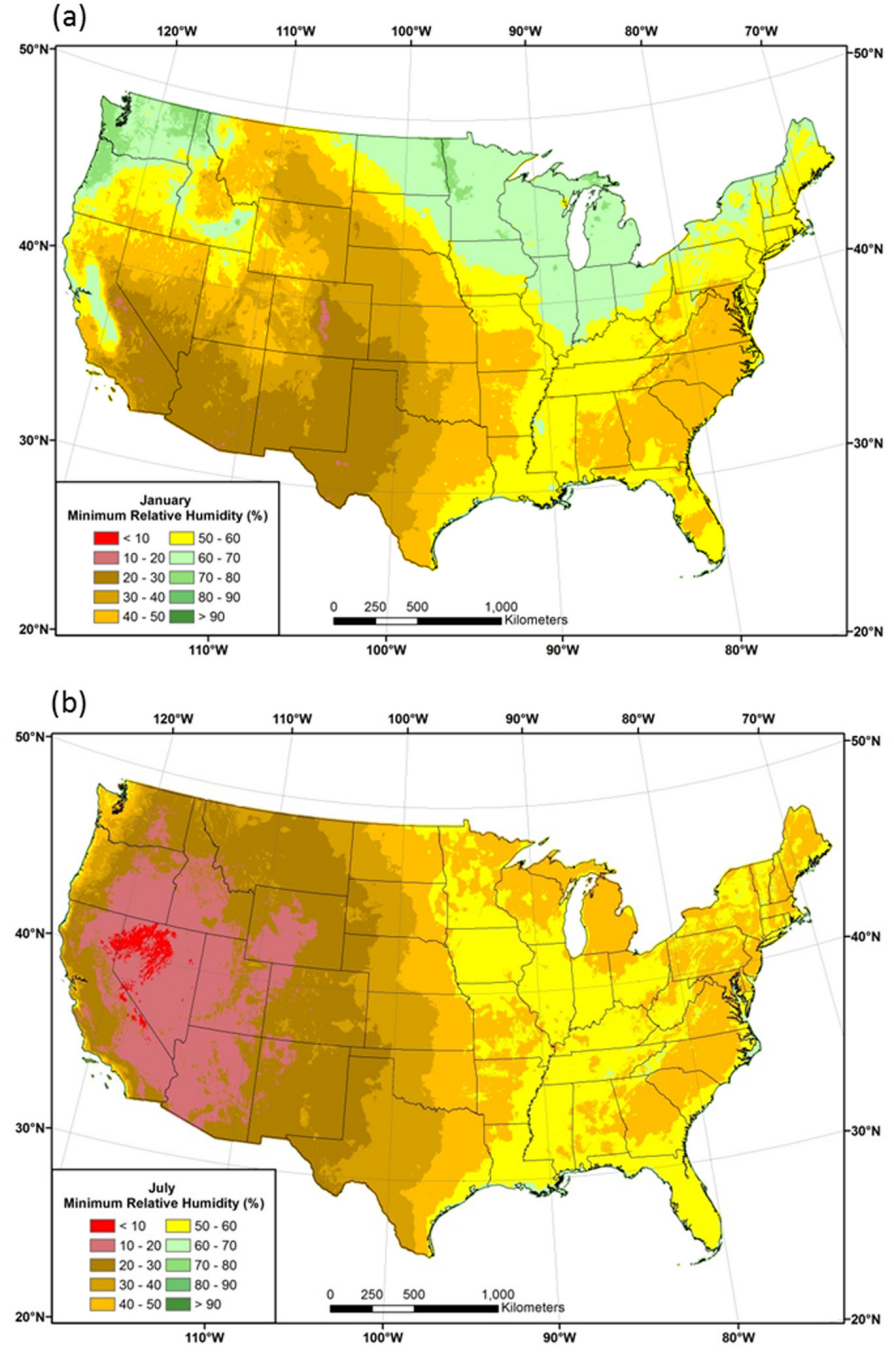
Maps of minimum relative humidity. Conterminous US 1981–2010 mean minimum relative humidity (*RH*_*min*_) estimated from mean maximum temperature (*T*_*max*_) and mean maximum vapor pressure deficit (*VPD*_*max*_) for (a) January and (b) July.

Maps of estimated 1981–2010 mean January and July *RH*_*min*_, estimated from grid values of *VPD*_*max*_ and *T*_*max*_, reveal some interesting features not easily seen in the maps of *VPD*_*max*_ (Fig 11). In January, *RH*_*min*_ values are as low as 10–15% on the lee side of the Rocky Mountains, associated with dry, Chinook (downslope) winds produced by a strong westerly jet stream. In contrast, *RH*_*min*_ values exceed 70% in the winter-wet Pacific Northwest. *RH*_*min*_ values are also above 70% in California’s Central Valley and Idaho’s Snake Plain, where extended periods of high pressure and calm winds promote temperature inversions that often result in persistent fog and low clouds (Fig 11a). Higher *RH*_*min*_ values in the upper Midwest reflect frequent cloudy weather during winter in this region.

*RH*_*min*_ patterns in July show a divided country, with a dry west and moist east (Fig 11b). The exception is the West Coast, where cool, moist onshore flow maintains relatively high humidity values throughout the day. The lowest *RH*_*min*_ (<10%) values are centered in Nevada, and more generally in the Great Basin. In the east, the highest values are found in regions that receive substantial moisture from the Gulf of Mexico, such as the southern Appalachians and Mississippi Valley.

### Uncertainty Analysis

Estimating the true errors associated with spatial climate data sets is difficult, and subject to its own set of errors [36]. This is because the true climate field is unknown, except at a relatively small number of observed points, and even these are subject to measurement and siting uncertainties (as has already been noted in the case of irrigated land, for example). Leave-one-out cross validation (C-V) approach is the most common evaluation method where each station is omitted from the dataset one at a time, the station value estimated in its absence, and the estimate and observation compared. The mean absolute error (MAE) and bias are typically calculated once the process is complete. While this approach is commonly used to assess error in interpolation studies, and is reported here, there are several disadvantages. An obvious disadvantage is that no error information is provided for locations where there are no stations. The single-deletion method favors interpolation models that heavily smooth the results, so that deletion of one station is relatively unimportant to the stability of the estimate. Randomly withholding a larger percentage of the station data at once can help to minimize this issue, as well as provide more robust error statistics. Withholding a stratified sample from the analysis is useful in detecting specific weaknesses or issues in the interpolation, as was done to investigate irrigated stations (also, see [36] for an example).

In this study, uncertanties in the mapped estimates were initially estimated by performing a C-V exercise with ASSAY, and the results compiled for each month for each of the three modeling regions (west, central, and east).

Even accounting for weaknesses in the C-V methodology, these errors are underestimates of the actual C-V uncertainty. One overlooked aspect of CAI is that it relies on predictor grids which have their own interpolation errors. These errors accumulate from one CAI generation to the next. For *T*_*dmean*_, uncertainties in the interpolated *T*_*min*_ predictor grids must be accounted for. In turn, *VPD*_*min*_ relies on interpolated *T*_*min*_ and *T*_*dmean*_, and *VPD*_*max*_ relies on *T*_*max*_, as well as *T*_*min*_ and *T*_*dmean*_.

To quantify the effects of error propagation on the CAI MAEs, the predictor grid interpolation error was introduced at each CAI step by using ASSAY interpolated estimates at station locations in their absence instead of the predictor grid values, which already have those station values built in. For *T*_*dmean*_, *T*_*min*_ values for all stations used in the mapping of the *T*_*min*_ predictor grid were estimated in their absence using ASSAY, and estimates common to both the *T*_*min*_ and *T*_*dmean*_ station datasets used as the values of *T*_*min*_ in an ASSAY C-V exercise for *T*_*dmean*_. *T*_*min*_ values for stations used in the interpolation of *T*_*dmean*_ but not *T*_*min*_, such as those from the SCAN and COAGMET networks, could not be estimated by ASSAY, because they were not used to create the predictor grid. However, they could still be included in the *T*_*dmean*_ C-V error estimation because, by definition, the estimates at their location on the *T*_*min*_ predictor grid were made in their absence.

For *VPD*_*min*_, ASSAY estimates of station *T*_*dmean*_ values, predicted using ASSAY estimates of *T*_*min*_ as described above, were used in combination with ASSAY estimates of *T*_*min*_ to form station values of first-guess *VPD*_*min*_, used as the predictor in the interpolation of *VPD*_*min*_. The result was station values of first-guess *VPD*_*min*_ that accounted for errors in the interpolation of both *T*_*min*_ and *T*_*dmean*_. These were used as the values of first-guess *VPD*_*min*_ in a C-V assessment of *VPD*_*min*_ with ASSAY. *VPD*_*max*_ involved the same steps as *VPD*_*min*_, except that first-guess *VPD*_*max*_ values were formed from a combination of ASSAY estimates of *T*_*max*_ and *T*_*dmean*_. Again, a C-V exercise for *VPD*_*max*_ was performed with ASSAY.

**Fig 12:**
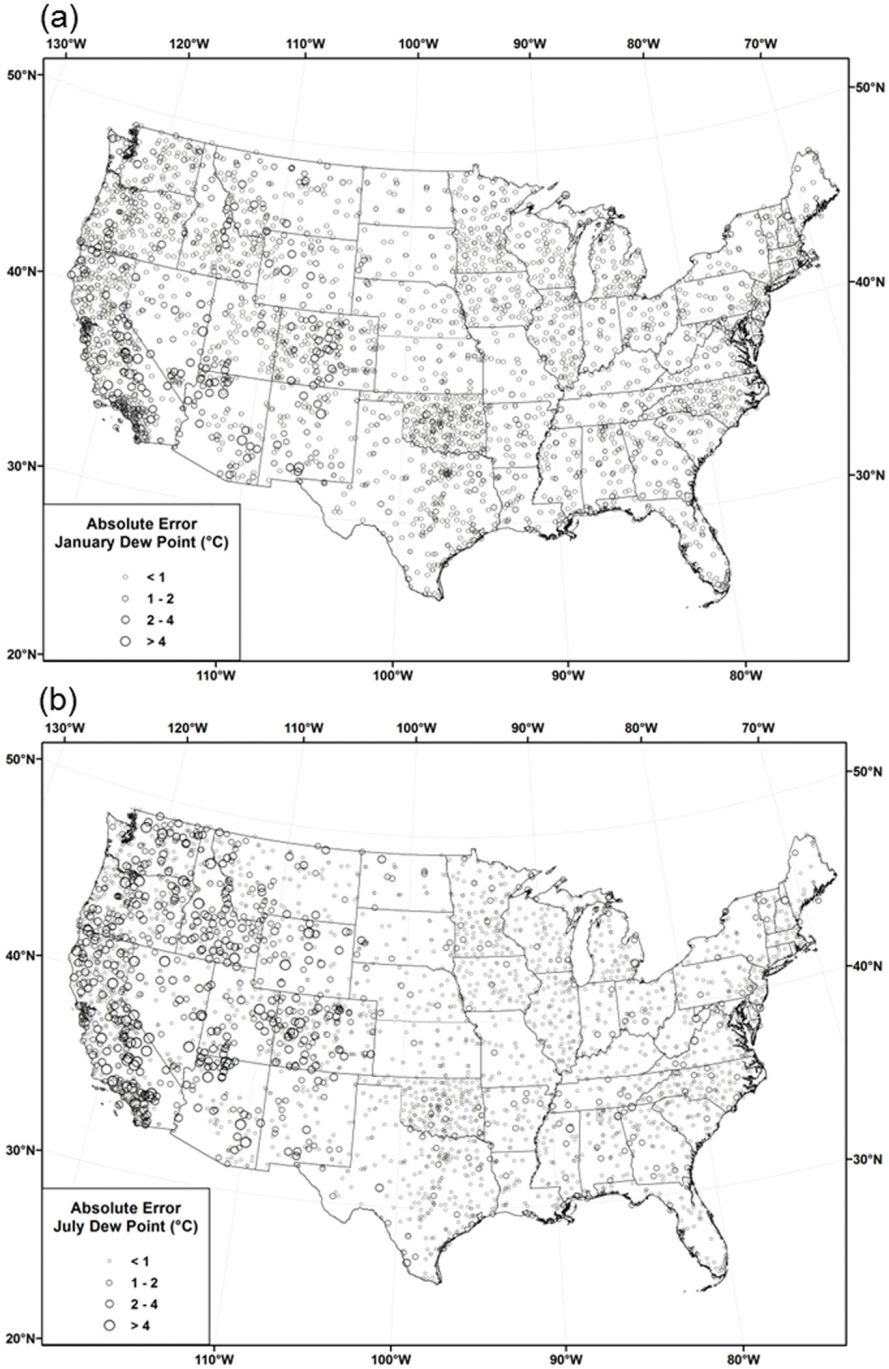
Maps of *T*_*dmean*_ absolute cross-validation errors. Errors for 1981–2010 mean monthly *T*_*dmean*_ in (a) January and (b) July.

**Fig 13:**
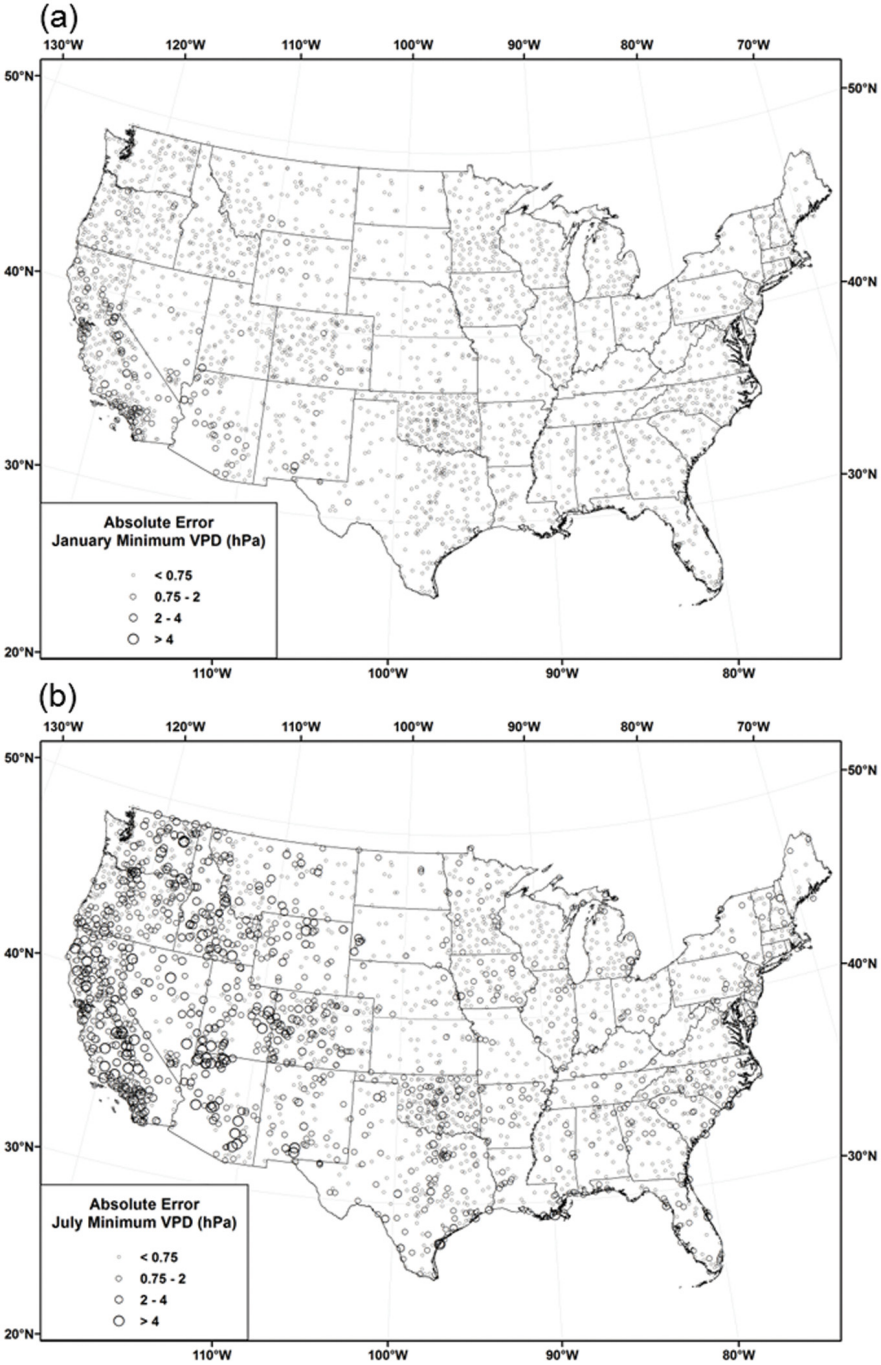
Maps of *VPD*_*min*_ absolute cross-validation errors. Errors for 1981–2010 mean monthly *VPD*_*min*_ in (a) January and (b) July.

**Fig 14:**
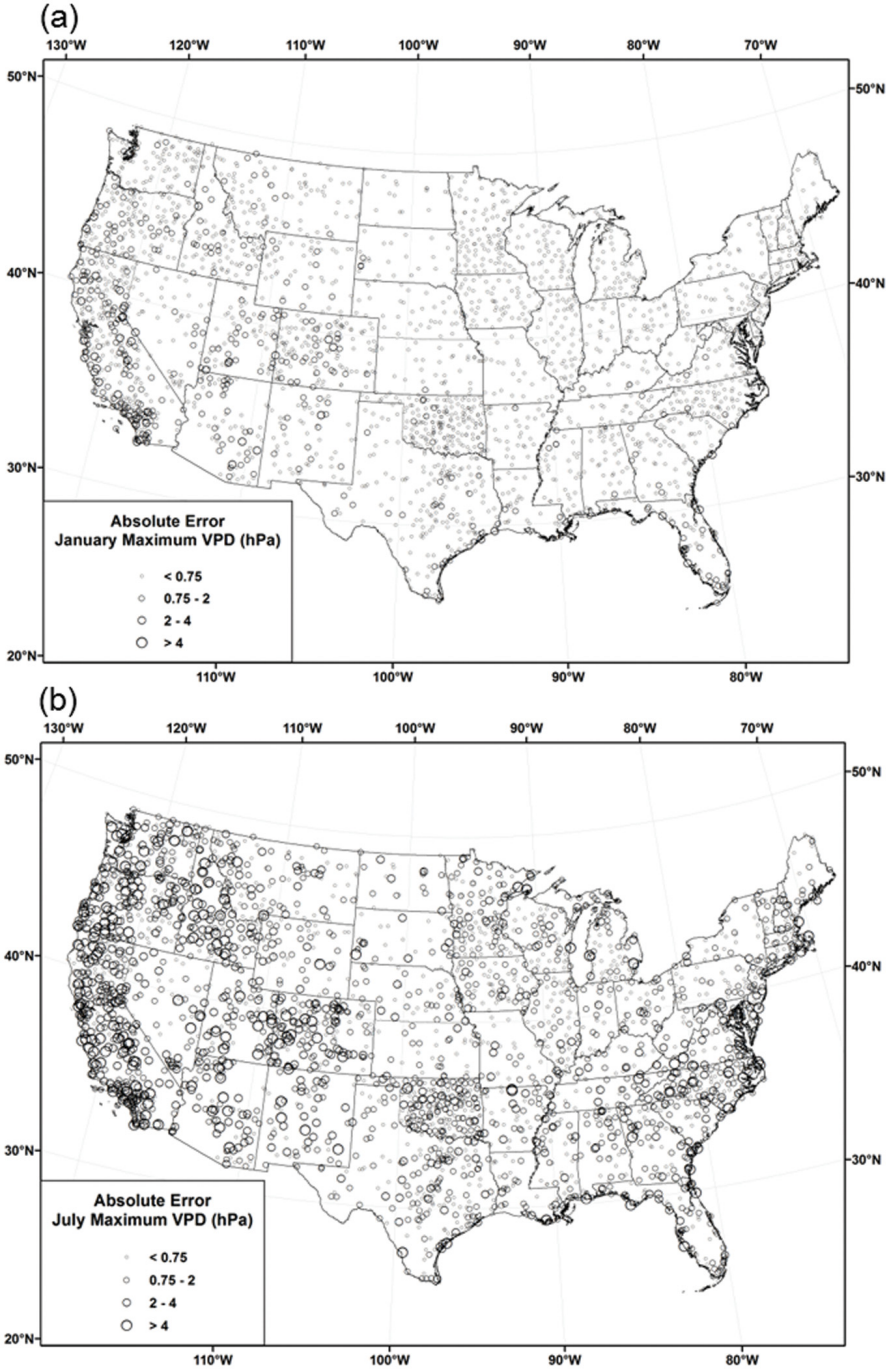
Maps of *VPD*_*max*_ absolute cross-validation errors. Errors for 1981–2010 mean monthly *VPD*_*max*_ in (a) January and (b) July.

Table 6 reports monthly ASSAY cross-validation MAEs for each of the modeling regions. MAEs that account for CAI interpolation error propagation are denoted with a CAI or CAI2. Figs 12, 13 and 14 show the distribution of cross-validation absolute errors at stations across the country for *T*_*dmean*_, *VPD*_*min*_, and *VPD*_*max*_, respectively.

**Table 6:** PRISM monthly cross-validation mean absolute errors (MAEs). Both interpolation-only MAEs, and MAEs accounting for uncertainties in the predictor grids (denoted by CAI or CAI2), are given below. See text for details.

Region/Variable	Month											
	Jan	Feb	Mar	Apr	May	Jun	Jul	Aug	Sep	Oct	Nov	Dec
**West**												
*T*_*max*_ (°C)	0.65	0.63	0.63	0.64	0.64	0.66	0.69	0.68	0.66	0.62	0.57	0.61
*T*_*min*_ (°C)	0.98	0.93	0.85	0.82	0.87	0.97	1.08	1.09	1.11	1.04	0.95	0.96
*T*_*dmean*_ (°C)	0.69	0.65	0.66	0.69	0.76	0.84	0.92	0.91	0.87	0.75	0.70	0.69
*T*_*dmean*_ CAI (°C)	0.76	0.75	0.77	0.79	0.88	1.00	1.12	1.10	1.03	0.86	0.80	0.79
*VPD*_*min*_ (hPa)	0.24	0.19	0.23	0.29	0.41	0.60	0.79	0.76	0.61	0.41	0.27	0.19
*VPD*_*min*_ CAI2 (hPa)	0.31	0.28	0.36	0.45	0.65	0.89	1.25	1.17	0.99	0.64	0.41	0.28
*VPD*_*min*_ (%)	41.9	31.2	32.2	31.0	28.5	30.1	30.5	32.1	32.2	29.2	34.0	33.7
*VPD*_*min*_ CAI2 (%)	58.5	45.2	50.0	45.8	42.9	45.8	48.3	48.7	52.0	44.7	51.3	49.7
*VPD*_*max*_ (hPa)	0.50	0.49	0.59	0.69	0.89	1.21	1.47	1.45	1.17	0.76	0.52	0.43
*VPD*_*max*_ CAI2 (hPa)	0.57	0.58	0.70	0.82	1.10	1.46	1.86	1.81	1.43	0.97	0.63	0.52
*VPD*_*max*_ (%)	11.1	8.7	7.4	6.4	6.1	6.3	6.0	5.9	5.8	5.8	7.6	10.1
*VPD*_*max*_ CAI2 (%)	11.8	10.2	8.4	7.0	6.8	6.8	6.9	6.9	6.5	6.7	8.5	11.4
**Central**												
*T*_*max*_ (°C)	0.44	0.46	0.48	0.48	0.46	0.44	0.44	0.43	0.42	0.41	0.38	0.40
*T*_*min*_ (°C)	0.65	0.63	0.55	0.55	0.53	0.53	0.55	0.58	0.63	0.67	0.64	0.63
*T*_*dmean*_ (°C)	0.45	0.42	0.40	0.42	0.42	0.45	0.45	0.44	0.43	0.41	0.41	0.42
*T*_*dmean*_ CAI (°C)	0.47	0.44	0.43	0.45	0.45	0.48	0.48	0.48	0.46	0.43	0.43	0.44
*VPD*_*min*_ (hPa)	0.11	0.12	0.15	0.20	0.28	0.37	0.45	0.43	0.34	0.23	0.16	0.11
*VPD*_*min*_ CAI2 (hPa)	0.13	0.15	0.19	0.27	0.37	0.49	0.60	0.56	0.46	0.32	0.21	0.15
*VPD*_*min*_ (%)	22.4	22.0	20.3	19.3	22.8	23.6	29.6	33.0	31.3	28.4	23.3	23.2
*VPD*_*min*_ CAI2 (%)	28.5	28.8	26.4	26.5	31.9	33.4	42.0	47.0	44.0	38.7	31.2	29.9
*VPD*_*max*_ (hPa)	0.24	0.28	0.37	0.50	0.64	0.75	0.90	0.86	0.67	0.42	0.30	0.24
*VPD*_*max*_ CAI2 (hPa)	0.29	0.34	0.43	0.62	0.79	0.96	1.13	1.07	0.84	0.55	0.37	0.30
*VPD*_*max*_ (%)	5.3	5.1	4.2	3.8	3.9	3.6	3.8	3.7	3.5	3.2	3.7	5.1
*VPD*_*max*_ CAI2 *(*%)	6.3	6.1	5.0	4.7	4.7	4.6	4.7	4.5	4.4	4.1	4.5	6.1
**East**												
*T*_*max*_ (°C)	0.40	0.46	0.49	0.51	0.50	0.49	0.48	0.47	0.43	0.42	0.37	0.37
*T*_*min*_ (°C)	0.62	0.64	0.61	0.65	0.65	0.60	0.60	0.63	0.69	0.71	0.64	0.61
*T*_*dmean*_ (°C)	0.46	0.42	0.40	0.40	0.40	0.40	0.38	0.38	0.40	0.39	0.39	0.39
*T*_*dmean*_ CAI (°C)	0.46	0.43	0.42	0.43	0.42	0.42	0.41	0.41	0.44	0.42	0.41	0.40
*VPD*_*min*_ (hPa)	0.10	0.12	0.19	0.25	0.31	0.36	0.38	0.38	0.33	0.24	0.18	0.13
*VPD*_*min*_ CAI2 (hPa)	0.13	0.15	0.23	0.32	0.39	0.48	0.50	0.48	0.41	0.30	0.22	0.15
*VPD*_*min*_ (%)	25.0	26.5	28.7	29.0	35.1	40.1	42.5	47.5	54.0	43.4	32.3	29.3
*VPD*_*min*_ CAI2 (%)	33.2	33.9	36.7	40.1	51.9	63.7	77.8	78.9	78.9	64.4	42.8	38.1
*VPD*_*max*_ (hPa)	0.25	0.28	0.50	0.59	0.75	0.93	1.01	0.91	0.73	0.48	0.34	0.26
*VPD*_*max*_ CAI2 (hPa)	0.28	0.32	0.45	0.69	0.89	1.07	1.23	1.12	0.89	0.59	0.40	0.30
*VPD*_*max*_ (%)	6.1	5.4	5.1	4.9	5.1	5.2	5.4	5.1	4.7	4.2	4.6	5.8
*VPD*_*max*_ CAI2 (%)	6.6	5.7	5.5	5.6	5.9	6.1	6.6	6.3	5.9	5.1	5.2	6.3

#### Dew Point

In general, *T*_*dmean*_ interpolation errors in the west were greater than those in the central and eastern US, due primarily to terrain-induced complexities in the vertical distribution of moisture and temperature. This is evidenced by higher absolute errors in the Rocky Mountains, Cascades, and Sierra Nevada (Fig 12). Regionally, MAEs for *T*_*dmean*_, interpolated as *DPD* (*T*_*min*_−*T*_*dmean*_), were less than 1°C in the west and less than 0.5°C in the central and east (Table 6). CAI MAEs that accounted for error propagation were 0.1–0.2°C larger than those that did not in the west, but less than 0.05°C larger in the central and east, owing to larger *T*_*min*_ interpolation errors in the west.

**Fig 15:**
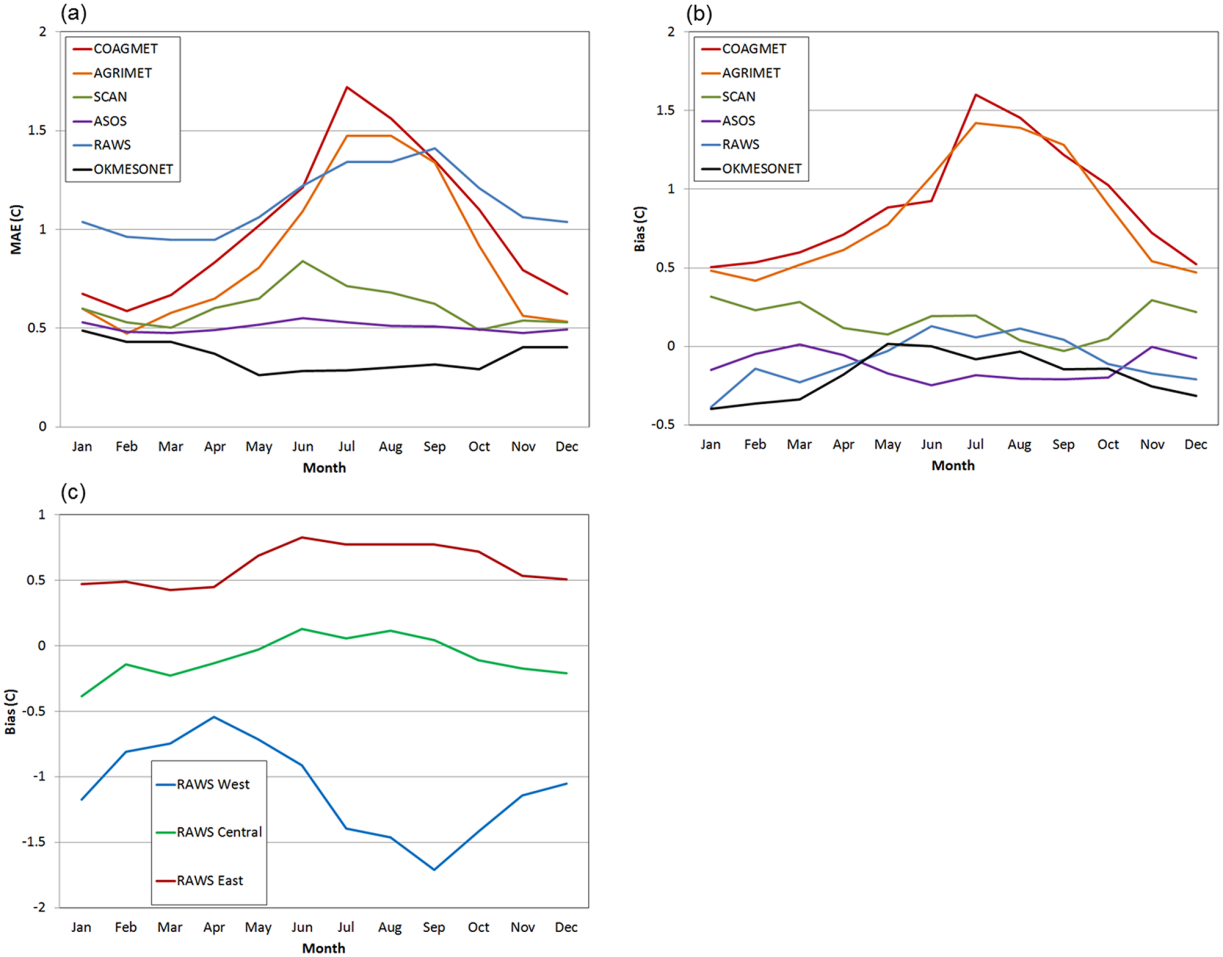
Network cross-validation MAE and bias of mean dew point depression (*DPD*). By month, station network, and region, when each network is entirely eliminated from the dataset at one time: (a) MAE for each network in the central region, except for AGRIMET, which is in the western region; (b), same as panel a, except showing bias; and (c) RAWS network bias for each region. The number of stations in each network is given in Table 1.

The *T*_*dmean*_ C-V analysis showed that the MAEs were inflated by systematic positive biases (lower observed *DPD* and higher *T*_*dmean*_ than predicted) in the COAGMET and AGRIMET networks. In fact, bias accounted for nearly all of the MAE. As discussed previously, stations in these networks were typically sited in or near irrigated fields for use in water management calculations. Despite subjective omission of stations producing the largest spatial inconsistencies, network-wide biases were still noticeable. Fig 15a and 15b show monthly *T*_*dmean*_ MAE and bias for each of the major station networks when each network is entirely eliminated from the dataset at one time. Plots are for the central region, to better control for the effects of complex physiographic features on interpolation bias; the exception is AGRIMET, which operates in the western region only, but stations are in flat agricultural areas, so interpolated predictions should be otherwise relatively unbiased. The peak MAE and bias in mid-summer correspond to the maximum difference in atmospheric moisture content one would be expect between irrigated and non-irrigated land. At their summer peaks, the COAGMET MAE and bias were 1.7 and 1.6°C, respectively. Peak AGRIMET MAE and bias were 1.5 and 1.4°C, respectively. These are in contrast to RAWS, for which the peak MAE was 1.4°C but the bias was only 0.1°C in the central region. The MAEs for these networks were much larger than the overall MAE for the central region of less than 0.5°C (Table 6).

A second source of systematic bias was found regionally in RAWS stations (Fig 15c). In the western region, biases were negative (higher observed *DPD* and lower *T*_*dmean*_ than predicted). In this region, the culprits may have been both local land cover and location. RAWS stations in the West are used primarily for fire weather applications, and are typically sited in open, ventilated areas away from transpiring vegetation. In addition, many RAWS stations are located on exposed terrain above locally humid cold air pools, and in foothill locations, which can be above larger-scale valley inversions, where a dry, free atmosphere aloft overlies relatively moist air below. Spring is the time of year when good vertical mixing causes a reduction in the incidence of inversions and cold air pools, which may explain the minimal bias at this time of year. The case for siting and location as causes for bias appears to be supported in the central region, where RAWS biases are near zero (Fig 15c). It is not clear why biases become somewhat positive (lower observed *DPD* and higher *T*_*dmean*_ than predicted) in the eastern region. One explanation may be that RAWS stations, often located in heavily forested areas in the east, are sampling more locally humid environments than other networks located at airports and in developed areas. The seasonal maximum bias in summer would correspond with the time of maximum transpiration from vegetation.

#### Vapor Pressure Deficit

MAEs for *VPD*_*min*_ and *VPD*_*max*_ were calculated regionally as percentages and in absolute hPa units (Table 6) and absolute errors mapped across the country (Figs 13 and 14). MAEs in absolute units were typically larger in summer than in winter, in keeping with characteristically larger vapor pressure deficits in summer due to higher temperatures (Table 6). Regionally, *VPD*_*min*_ MAEs were less than 1 hPa in all months and regions, owing to relatively small absolute values. In January, the highest absolute *VPD*_*min*_ errors were concentrated in the desert southwest, where moisture deficits are greatest (Fig 13). These higher errors spread to most of the west in July, when deficits are seasonally high. Regional MAEs ranged from roughly 30–40 percent in the west, 20–30 percent in central, and 25–50 percent in the east. CAI2 MAEs that accounted for error propagation were 0.1–0.5 hPa and 15–20% larger in the west, 0.05–0.15 hPa and 5–15% larger in the central, and 0.05–0.15hPa and 10–25% larger in the east.

Regional *VPD*_*max*_ absolute MAEs were approximately double those for *VPD*_*min*_ in absolute units. Percentage MAEs were accordingly much lower, ranging from roughly 6–11, 3–5, and 4–6 percent in the west, central, and east, respectively. CAI2 MAEs that accounted for error propagation were 0.1–0.5 hPa and 0.5–1.5% larger in the west, and 0.05–0.25 hPa and 0.5–1.5% larger in the central and east. MAEs were again inflated by systematic positive biases (lower observed *VPD* observations than predicted) from COAGMET and AGRIMET. The spatial and temporal distributions of absolute errors were similar to that of *VPD*_*min*_, with the highest January errors in the west, spreading to the east in July (Fig 14). July *VPD*_*max*_ errors were higher in the eastern US than those of *VPD*_*min*_, because warm maximum temperatures in this region produce significant daytime vapor pressure deficits, despite relatively high *RH* values.

## Summary and Conclusions

Long-term normal grids of 1981–2010 mean monthly average daily *T*_*dmean*_, *VPD*_*min*_ and *VPD*_*max*_ were developed for the conterminous United States. The *T*_*dmean*_ grids update previous unpublished *T*_*dmean*_ normals used as CAI predictor grids in PRISM monthly time series datasets, and to our knowledge, the *VPD*_*min*_ and *VPD*_*max*_ normal grids are the first of their kind. Interpolation of the long-term monthly averages was performed using PRISM. Nearby stations entering the PRISM local regression functions (one per pixel) were assigned weights based on the physiographic similarity of the station to the grid cell that included the effects of distance, elevation, coastal proximity, vertical atmospheric layer, and topographic position. Relatively few stations were available for these variables, prompting us to use CAI to improve interpolation accuracy. In the CAI process, 1981–2010 monthly *T*_*min*_ grids served as predictor grids for the interpolation of *T*_*dmean*_, expressed as the dew point depression (*DPD* = *T*_*min—*_ *T*_*dmean*_). Second-generation CAI (CAI2) involving *T*_*dmean*_ and *T*_*min*_, and *T*_*dmean*_ and *T*_*max*_, predictor grids were used to interpolate *VPD*_*min*_ and *VPD*_*max*_, respectively.

The general patterns of *T*_*dmean*_ were similar to those of *T*_*min*_ in both winter and summer. However, the assumption that *T*_*dmean*_ is equal to *T*_*min*_ did not hold over large parts of the country. In winter, *T*_*dmean*_ was several degrees higher than *T*_*min*_ in the northern US and in cold valley locations of the West. In summer, *T*_*dmean*_ averaged 10–25°C lower than *T*_*min*_ in the desert southwest. *VPD*_*min*_ was very low over most of the country, due to a combination of low temperatures and relatively small differences between ambient and dew point temperatures in the morning hours. Patterns of *VPD*_*max*_ roughly followed those of *T*_*max*_ in winter, with the lowest values in the northern tier and western mountains, and the highest in the southern states. In summer, the area of high *VPD*_*max*_ expanded considerably, reaching a maximum of over 60 hPa in the desert southwest. Coastal regions of the West exhibited relatively low *VPD*_*max*_ values, where cool, marine air penetrates inland.

A PRISM interpolation uncertainty analysis was performed using C-V exercises. Since CAI relies on predictor grids which have their own interpolation errors, these errors were included in the overall error estimates. To quantify the effects of error propagation on the CAI MAEs, the predictor grid interpolation error was introduced at each step by using ASSAY predictions at station locations instead of the interpolated predictor grid values. When accounting for error propagation, MAEs for *T*_*dmean*_ were 0.8–1.1°C in the west modeling region and less than 0.5°C in the central and east, with the greatest errors typically occurring in summer. *VPD*_*min*_ MAEs were generally less than 1 hPa in all months and regions, and *VPD*_*max*_ MAEs about double those values.

Overall, accounting for CAI error propagation increased C-V errors, but not as much as expected, especially when compared to the initial MAEs associated with the *T*_*max*_ and *T*_*min*_ predictor values. One reason for this appears to be that the errors were not systematically biased in one direction; that is, overestimates and underestimates partly canceled each other, producing lower net error increases.

Some stations in the AGRIMET and COAGMET networks were found to have consistently high *T*_*dmean*_ and low *VPD* values during the summer months. Stations in these networks were typically sited in or near irrigated fields for use in water management calculations, resulting in more humid conditions than locations distant from irrigated areas. This raised questions about whether the grids should represent conditions over irrigated land. A middle ground was taken, where a few stations causing the most severe spatial discrepancies were omitted from the datasets. In addition, the siting of RAWS stations was posited as the reason for lower than expected humidity in the west and higher than expected in the east. These issues highlight the effect that local topographic position and land use/land cover properties can have on the spatial patterns of atmospheric moisture content and deficit.

In combination with existing PRISM grids of *T*_*min*_ and *T*_*max*_, grids of *T*_*dmean*_, *VPD*_*min*_ and *VPD*_*max*_ allow the user to derive many other atmospheric moisture variables, such as minimum and maximum *RH*, vapor pressure, and *DPD*. Accompanying assumptions may need to be made, however, such as those outlined in the derivation of *RH*_*min*_ and *RH*_*max*._ These new grids will serve as the predictor grids in second-generation CAI to produce an updated version of the PRISM *T*_*dmean*_ monthly time series dataset that covers the years 1895-present [9], and initial versions of monthly *VPD*_*min*_ and *VPD*_*max*_ time series grids for the same period using third-generation CAI. Similar methods will be used to produce initial versions of daily *T*_*dmean*_, *VPD*_*min*_ and *VPD*_*max*_ time series datasets, spanning 1981-present. All of the PRISM normal grids discussed here are available online at http://prism.oregonstate.edu, and include 800-m and 4-km resolution data, images, metadata, pedigree information, and station inventory files. Links to the papers cited in Table 3 and elsewhere are also available from this website.
